# Longitudinal study assessing antimicrobial usage in broiler production cycles in northern Vietnam in 2022 to 2023

**DOI:** 10.1016/j.psj.2026.107918

**Published:** 2026-10-06

**Authors:** Chen Xin, Trang Nguyen, Quoc Hung Nguyen, Saira Butt, Huong Quynh Luu, Hoa Thi Thanh Pham, Anne Conan, Ngoc Thi Pham, Dirk Pfeiffer, Damer Blake, Fiona Tomley, Priscilla F. Gerber, Beatriz Calvo-Urbano, Guillaume Fournié, Ludovic Pelligand

**Affiliations:** a Centre for Applied One Health Research and Policy Advice (OHRP), City University of Hong Kong, Hong Kong SAR, China; b Department of Infectious Diseases and Public Health, Jockey Club College of Veterinary Medicine and Life Sciences, City University of Hong Kong, Hong Kong SAR, China; c Vietnam Institute of Animal and Veterinary Science (VIAVS), Ha Noi, Vietnam; d Department of Pathobiology and Population Sciences, The Royal Veterinary College, UK; e French Agricultural Research Centre for International Development (CIRAD), Ha Noi, Vietnam; f French Agricultural Research Centre for International Development (CIRAD), UMR ASTRE, Harare, Zimbabwe; g Animals, Health, Territories, Risks, Ecosystems (ASTRE), Univ Montpellier, CIRAD, INRAE, Montpellier, France; h INRAE, VetAgro Sup, UMR EPIA, Université de Lyon, Marcy I’Étoile, France; i INRAE, VetAgro Sup, UMR EPIA, Université Clermont Auvergne, Saint Genès Champanelle, France; j Comparative Biomedical Sciences, The Royal Veterinary College, London, UK; k Clinical Services and Sciences, The Royal Veterinary College, London, UK

**Keywords:** Antibiotic, Chicken, Prophylactic, Therapeutic, Mortality

## Abstract

Antimicrobial drugs (AMDs) are commonly used in chicken production in Vietnam for both preventive (prophylactic) and therapeutic purposes. Characterizing how the frequency of antimicrobial usage (AMU) changes from flock placement to harvest, varies by purpose, and may be influenced by production stage and mortality levels, can guide antimicrobial stewardship interventions to mitigate the risk of antimicrobial resistance (AMR) in broiler production.

We investigated daily AMU and mortality patterns in production cycles of slow-growing broiler chicken flocks raised on 60 farms in two provinces (Ha Noi and Bac Ninh) in northern Vietnam at the end of 2022 and the beginning of 2023. The association between the daily probability of AMU and mortality risk was evaluated using generalized additive mixed models (GAMMs). The dynamic process of initiating a new treatment, and the expected duration of that episode, were further assessed using a two-state semi-Markov process (SMP) within a Bayesian framework.

A total of 569 treatment events (70.5% preventive vs. 29.5% therapeutic) were recorded across all 60 monitored cycles. A median value of 186.1 mg/Population Correction Unit (PCU) AMDs was used for prophylaxis, while 116.7 mg/PCU was used for therapeutic treatment per production cycle. The most frequently administered AMDs for preventive treatments were colistin (23.2%), amoxicillin (19.2%), and doxycycline (11.7%). Therapeutic treatments mostly involved doxycycline (26.8%), florfenicol (17.9%), and amoxicillin (13.7%). The main effect of within-flock mortality was not significant, but the interaction between mortality and broiler age was associated with AMU both in the case of preventive and therapeutic AMU (p-value <0.05), suggesting that the effect of mortality on AMU may have varied depending on the broiler age. Broiler age was strongly and non-linearly associated with AMU (p<0.05), with distinct temporal patterns for preventive and therapeutic use. At the start of a production cycle, initiation of preventive treatment was substantially more likely than initiation of therapeutic treatment (day 0: risk ratio [RR] = 8.6), and prophylactic treatment remained dominant during the early cycle (RR > 2 before day 22). These findings indicate that preventive treatment is the primary driver of AMU during the early cycle, highlighting the need for stewardship interventions at this stage, focused on reducing non-essential prophylactic use and promoting lower-risk alternatives.

## Introduction

Broiler farming is one of the most intensive livestock farming systems in the world, and chicken is the most widely consumed type of meat (Averós and Estevez, 2018; Organisation for Economic Co-operation and Development (OECD), 2018). Globally, most commercial chicken production occurs in high-density systems that utilize a small number of fast-growing chicken lines that typically reach market size between 30 and 47 days of age (Henuk and Bailey, 2017; Mottet and Tempio, 2017). However, production systems differ markedly across countries, and in Vietnam around 72% of the national broiler flock are slow-growing broilers (Thi Dien et al., 2023; Xin et al., 2025). These are generally colored-feather hybrids of commercial broilers and local breeds and are raised for periods of ∼70 days to several months. They are raised in semi-intensive systems that combine traditional husbandry practices with adaptations of more intensive technologies and management practices. These systems include small- to medium-sized flocks (with flock size ranging from 51 to 2,000 birds) that are kept in enclosures or allowed access to outdoor areas, such as backyards and orchards, and they supply meat and live birds to local markets (Burgos et al., 2007; M Y Birhanu et al., 2021).

Throughout a production cycle, antimicrobial drugs (AMDs) may be administered therapeutically to entire flocks of chickens after clinical signs of disease are observed in one or more birds (Hofacre et al., 2013). Although the use of AMDs to promote growth in animal production systems has been prohibited in Vietnam since 2018 (Ministry of Agriculture and Rural Development, 2016), their use for disease prevention (prophylaxis), defined as the administration of AMDs to apparently healthy birds at risk of infection or prior to the onset of clinical disease (World Health Organization, 2017), has remained permitted until 2026 (Vietnamese Government, 2020). A literature review documenting AMU in livestock in Indonesia, Thailand, and Vietnam indicated that disease prevention is a primary driver of antimicrobial usage (AMU), particularly in broiler production systems (Coyne et al., 2019). In Vietnam, AMU is high in broiler production, both in the frequency and quantities of drugs used, and it was recently reported that 87.9% of poultry farmers used AMDs (Luu et al., 2021). In the Mekong Delta, levels of drug usage expressed as “the amount of AMDs used to produce one broiler chicken” were approximately six-fold higher than those reported in some European countries, with 84% of AMD used for prophylaxis rather than therapy (Carrique‐Mas et al., 2015). Such high levels of AMU may be driven by a high pathogen burden and associated mortality risks (Adam et al., 2019; Emes et al., 2023). Other contributing factors may include easy access to relatively inexpensive over-the-counter AMDs through a dense network of veterinary drugstores, as well as lack of farmers’ knowledge of responsible AMU and disease prevention (Bâtie et al., 2022; Truong et al., 2019).

High AMU can promote the selection of antimicrobial-resistant bacteria (Khan et al., 2024; Mughini-Gras et al., 2022), with the potential for subsequent spread of resistant bacteria and/or resistance genes amongst chickens and spillover to other species including humans (European Medicines Agency (EMA), 2020). Vietnam has a policy framework to improve antimicrobial stewardship in livestock production, framed within a National Action Plan (Minister of Ministry of Agriculture and Rural Development, 2017). One component of this plan emphasizes the need to generate additional evidence on AMU, including through farm-level surveys to quantify AMU patterns.

Despite its policy relevance, assessments of AMU in broiler farming in Vietnam and other low- and middle-income countries have mostly relied on cross-sectional designs, in which data are collected at a single time point to identify usage patterns and associated factors (Ibrahim et al., 2023; Kemunto et al., 2026; Luu et al., 2021; Mahmood et al., 2024; Umair et al., 2021). Such designs cannot reliably capture the timing, duration, or sequence of antimicrobial administrations during a production cycle, being vulnerable to recall bias, particularly when farmers are asked to report non-recent treatments. In contrast, longitudinal designs follow the same flocks over time, allowing AMU to be quantified across the entire cycle, its temporal dynamics to be described, and its relationship with mortality and treatment purpose to be assessed. These kinds of studies can identify periods when prophylactic use is more likely than therapeutic use and when stewardship interventions may be most relevant. For example, a longitudinal study in the Mekong Delta identified the early brooding phase as a period associated with disease, mortality, and AMU in broiler production (Carrique-Mas et al., 2019). However, only a few longitudinal studies in southern Vietnam (Mekong Delta) have quantified AMU in slow-growing broilers (Cuong et al., 2021, Cuong et al., 2019), whilst the semi-intensive production systems in northern Vietnam remain understudied.

To address these gaps, we have conducted a longitudinal study of slow-growing broiler flocks on semi-intensive farms in northern Vietnam. At the production-cycle level, we quantified AMU using complementary weight- and dose-based metrics, based on data from farm daily drug records and information on drug packaging. The weight-based metric, expressed as milligrams of active ingredient per population correction unit (mg/PCU) (British Veterinary Poultry Association (BVPA), 2018), quantified the total amount of AMDs administered and facilitated comparison across studies and settings. Treatment-incidence metrics based on Used Daily Dose ($TI_{\text{UDD}}$) and Defined Daily Dose ($TI_{\text{DDD}}$) were used to characterize treatment frequency (Persoons et al., 2012) and facilitate comparison across AMDs with different recommended dosages. Reporting both TIUDD and TIDDD distinguishes treatment incidence calculated using actual on-farm doses from that calculated using a standardized reference dose. Daily AMU records were then used to assess treatment timing, examine associations between AMU, broiler age, and within-flock mortality, and identify periods during which prophylactic rather than therapeutic treatment was more likely to be initiated.

## Materials and methods

### Ethical Approval

This study was approved by the Ethical Review Board for Biomedical Research, Hanoi University of Public Health (Viet Nam) (020-433/DD-YTCC) and the Royal Veterinary College (UK) Ethics and Welfare Committee (URN: 2020 1983-3). All participants informedly agreed to participate in the study.

### Study Design, Study Area, Farm Selection

This longitudinal study was conducted in Ha Noi and Bac Ninh provinces (Fig. 1), between October 2022 and June 2023. These provinces are among the most densely populated in northern Vietnam, in terms of both human and poultry populations (General Statistics Office of Vietnam (GSO), 2023a, 2023b)

**Fig. 1:**
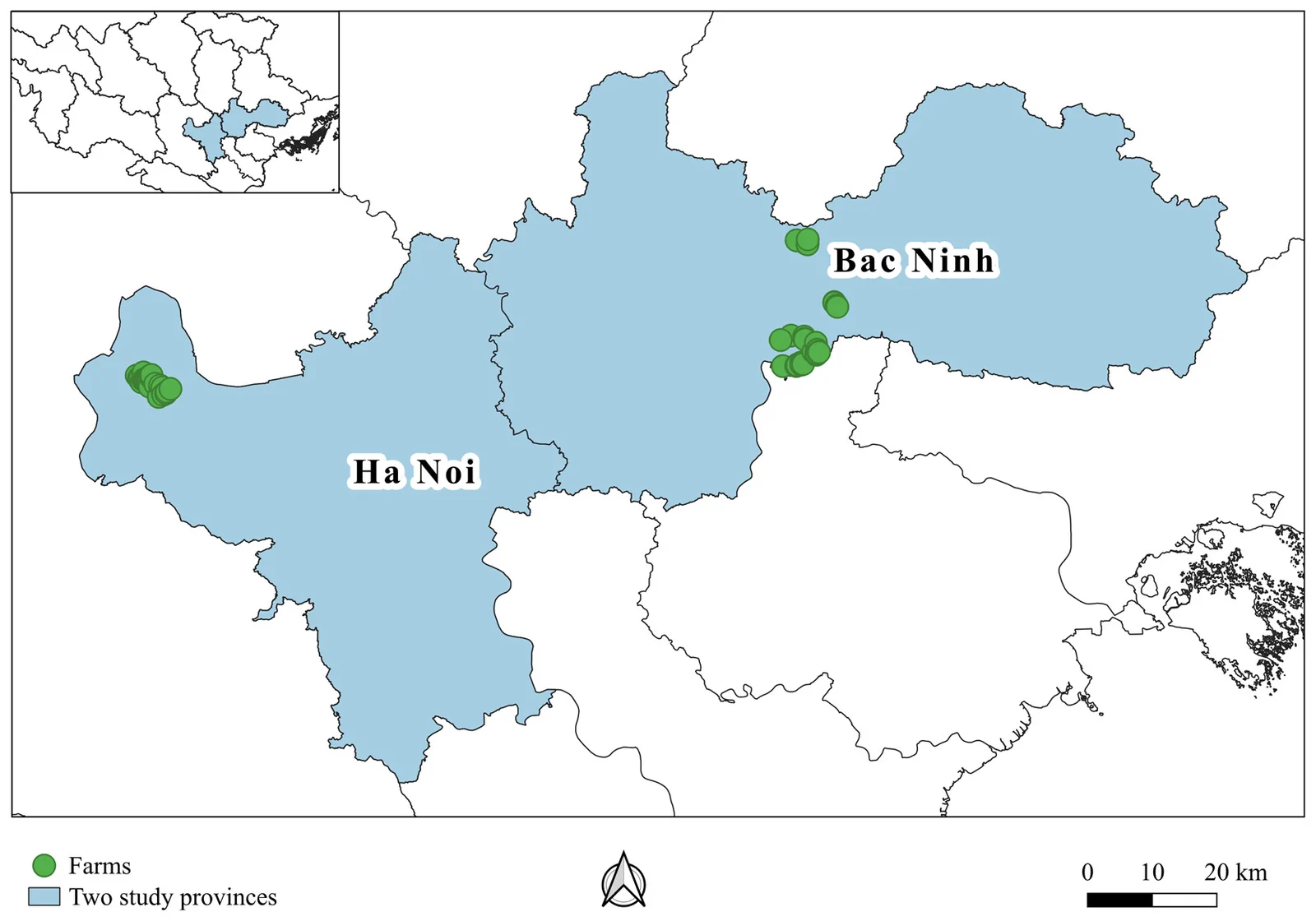
Location of sampling chicken farms (green points) in the two study provinces in Vietnam (base layer of the map source (General Statistics Office of Vietnam (GSO), 2026)).

Sixty slow-growing broiler farms were recruited and monitored over a single complete production cycle, from the placement of chicks on farms to their sale at adult age. This yielded 60 farm-production-cycle observations, with each monitored production cycle treated as the analytical unit. Farms were stratified by province. Eligible farms were required to rear flocks of between 1,000 and 5,000 slow-growing broilers, provide outdoor access, and operate production cycles lasting ≥70 days. Each cycle was treated as a study unit. Local officers from the Department of Animal Health identified all slow-growing broiler farms within each study area (one commune in Hanoi and one district in Bac Ninh) and collected information to confirm eligibility. Lists of eligible farms were then used as sampling frames. In each study area, 30 farms were randomly selected, with the sample size determined by budget and logistical considerations. All selected farms were visited, and oral consent to participate was obtained from the owner or manager of each farm.

### Data Collection

Before the start of each production cycle, researchers conducted an initial preparatory farm visit during which farmers were introduced to the study protocol and trained in the daily AMU recording procedures.

The number of chickens in each batch was recorded at the start of the production cycle, and farmers recorded daily death counts, sales, and water consumption for each monitored batch. Farmers also weighed three randomly selected chickens weekly. These data were reported to the research team through daily phone calls (Fig. 2). Each batch was visited four times. The initial visit occurred approximately during the second week, followed by a subsequent visit five to six weeks later. The third visit took place near the end of the cycle, before traders collected chickens from the batch. The last visit was conducted at the end of the trading period, just after the sales of the last chickens left from the batch. AMU data were collected from two sources, following (Ibrahim et al., 2023). First, farmers completed paper-based drug-use forms daily starting from chick placement, documenting the brand name of each AMD, the amount of drug product used per administration (farmers reported that any drug was given once per day), the route of administration (if given through water, the volume of water used for drug product dilution was recorded), and the stated purpose for use. Clinical signs were also recorded when therapeutic use was reported. These records were reported to our research team by phone every three days. Second, drug packaging records were collected by retrieving packages, packets, and bottles deposited by farmers in a dedicated on-farm collection box and collected by researchers during each visit, allowing documentation of AMD brand names, active substances and active ingredient in salt or hydrate, concentration, and product form (powder or solution) from package labels (Fig. 2). During each farm visit, drug container contents were retrieved, package labels were photographed, and data were uploaded to a dedicated Open Data Kit (ODK) platform (Muntashir and Arefin, 2017). All data were cleaned and exported as comma-separated values (CSV) files for further processing and analysis in R 4.4.3 (R Core Team 2025).

**Fig. 2:**
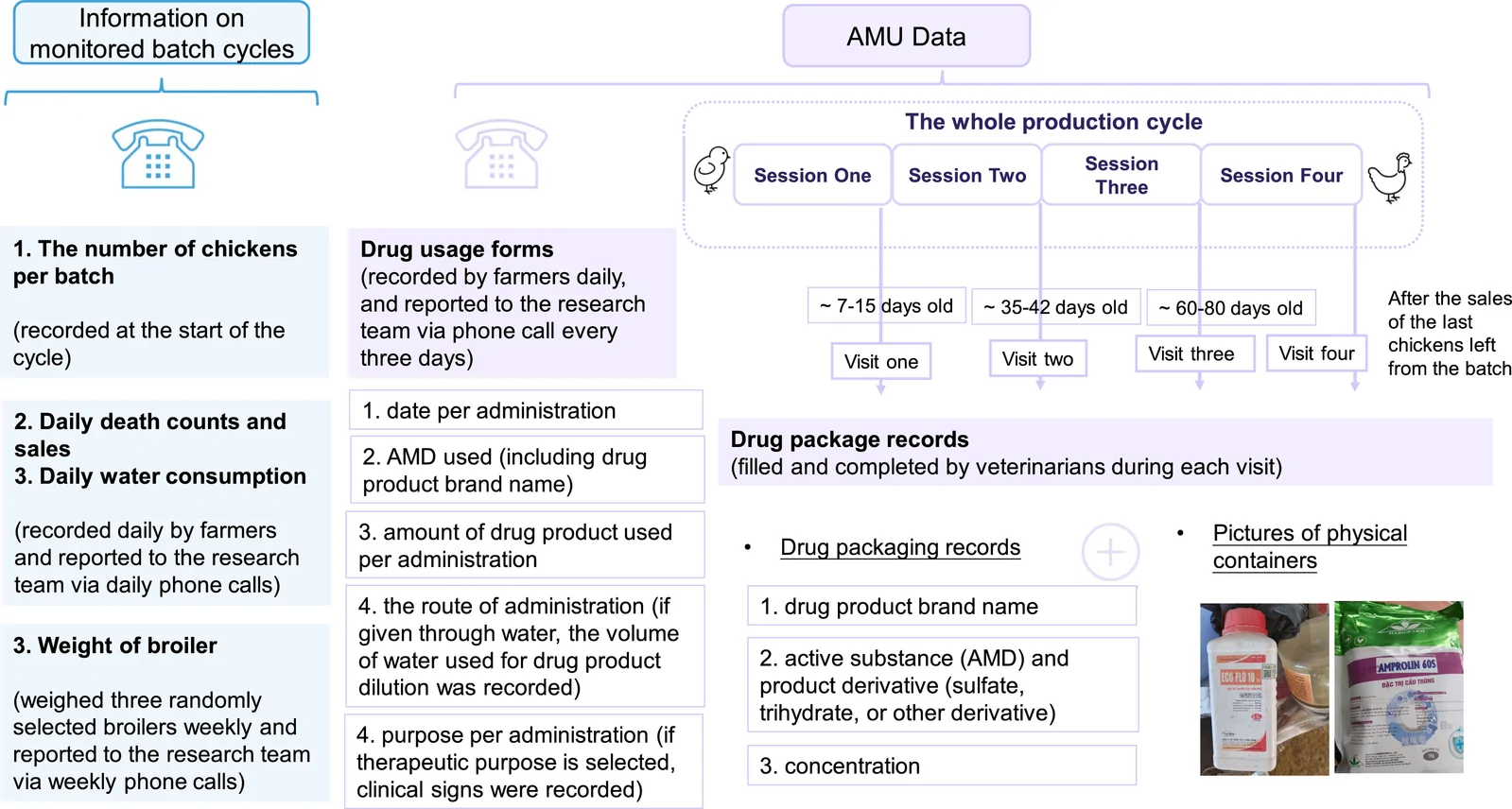
Information collected for monitored batches. AMDs were defined as antimicrobial substances authorized for use as human or veterinary medicines, identified from drug usage forms and drug package records. AMU refers to the daily usage status of AMDs, indicating whether a farm has a record of AMD use on any given day for any purpose.

### Data Handling

***Definition of Mortality at the Population and Batch Levels.*** For each monitored batch, the number of live broilers at the start of a given day was calculated as the initial flock size minus the cumulative number of deaths and sales recorded up to the previous day. The daily mortality risk over the entire production cycle was calculated as the ratio between the number of chickens that died on a given day and the number of live chickens at the start of that day, both at the population level across all farms and at the batch level for each monitored flock.

***AMD Data Integration and Variable Definition.*** AMDs were defined as antimicrobial substances authorized for use as human or veterinary medicines, classified into antimicrobial classes, under four categories according to the European Union (EU) Antimicrobial Advice ad hoc Expert Group (AMEG) based on their importance for human medicine and the risk of resistance transmission from animals to humans (European Medicines Agency (EMA), 2020). Data from drug usage forms and packaging records were first combined. Specifically, for each farm, all records from the daily drug usage forms were retained, compiled chronologically, and cross-checked against packaging records. When a discrepancy was identified, including cases in which the packaging information was incomplete or missing, farmers were consulted to confirm the active substance in antimicrobial-containing products, while active ingredients, concentrations and product forms were confirmed using photographs of package labels. The final integrated dataset was structured such that each entry represented a single AMD administration on a particular farm on a specific day. The integrated dataset was subsequently processed to define antimicrobial-substance treatment events and daily usage statuses. To capture treatment patterns, repeated administrations of the same AMD with the same dose, route, indication, and formulation on consecutive days within a given farm were grouped into a single antimicrobial-substance treatment event. A change in any of these characteristics resulted in a new treatment event. Each treatment event, therefore, comprised one or more consecutive-day administrations of the same AMD on the same farm, and was characterized by its timing, farmer-reported purpose - preventive (prophylactic) or therapeutic use - and its duration.

A ridgeline plot was generated to visualize the temporal distribution of treatment events for each recorded AMD throughout the production cycles. The x-axis represents the broiler age (in days) at the end of each treatment event. At the same time, the y-axis lists the specific AMD density, which illustrates the relative frequency of treatment events across different broiler ages. Meanwhile, the temporal trends in the daily proportion of farms reporting AMU by purpose were also plotted, with smoothed curves fitted using GAMMs with the 'gamm' function from the *mgcv* package (version 1.9-3) (Wood, 2000).

### AMU Quantification

Farm-level AMU, including both preventive and therapeutic use, was quantified using complementary weight- and dose- based metrics. On each treatment day, the adjusted Used Daily Dose (UDD) was first calculated from farm treatment records. These daily UDD estimates were used to derive the total active moiety of antimicrobials administered over the production cycle, which was standardized by the population correction unit (PCU) to estimate overall antimicrobial consumption as mg/PCU. Treatment frequency was quantified using treatment incidence based on UDD ($TI_{\text{UDD}}$) and the Defined Daily Dose ($TI_{\text{DDD}}$). As a preliminary step, antimicrobial products were standardized before calculating AMU metrics. For products where the active ingredient was reported as a salt or hydrate, the quantities were converted to the corresponding active moiety using a conversion factor (World Organisation for Animal Health (WOAH), 2023) (Table S1). The AMD concentration listed on each product label was therefore expressed as milligrams of active moiety per unit of the commercial product, such as mg/g for solid formulations or mg/mL for liquid formulations. For colistin, which was reported in International Units (IU) per unit of product (IU/mL or IU/g), an additional conversion step was performed to convert the content to milligrams of colistin base equivalents, using the international standard potency of colistin base, 30,000 IU/mg (Falagas and Kasiakou, 2006).

***Adjusted Used Daily Dose (UDD).*** For each AMD, UDD represents the amount of actual active moiety administered (mg) per kilogram of broiler body weight per day, expressed in mg/kg (Persoons et al., 2012). Since AMDs were administered orally via drinking water and feed, it is further adjusted based on actual water and feed consumption. For batches where daily water consumption was lower than the dilution volume, the UDD was calculated based on the actual water intake. Conversely, when water consumption exceeded the dilution volume, the UDD was determined using the dilution volume. Thus, the adjusted UDD was calculated as the mixing concentration of active moiety in mg/L of water, multiplied by the actual daily drinking-water consumption or dilution volume (L/day), divided by the total body weight of the treated broilers on the treatment day.

(1) $$\text{UD}D_{\text{adjust}\left(\text{water}\right)}=\frac{\;\text{active}\;\text{moiety}\;\text{concentration}\;\text{in}\;\text{water}\;\left(\frac{\text{mg}}{L}\right)\;\times \;\text{actual}\;\text{water}\;\text{intake}\;\text{or}\;\text{dilution}\;\text{volume}\;\text{on}\;\text{treatment}\;\text{day}\;\left(L\right)}{\text{numbers}\;\text{of}\;\text{broilers}\;\text{treated}\;\times \;\text{estimated}\;\text{chicken}\;\text{weight}\;\text{on}\;\text{treatment}\;\text{day}\;\left(\text{kg}\right)}$$

Where the AMD concentration was converted to milligrams of active moiety per liter of drinking water.

For drugs administered through feed, we assumed 100% feed consumption. The numerator for the amount of AMD (active moiety) administered on the treatment day was calculated as the converted drug concentration on product multiplied by the amount of product given on that day, expressed as:

(2) $$\text{UD}D_{\text{adjust}\left(\text{feed}\right)}=\frac{\text{amount}\;\text{of}\;\text{active}\;\text{moiety}\;\text{per}\;\text{unit}\;\text{of}\;\text{product}\;\left(\text{mg}\right)\times \;\text{the}\;\text{amount}\;\text{of}\;\text{product}\;\text{used}\;\text{per}\;\text{treatment}\;\text{day}\;}{\text{numbers}\;\text{of}\;\text{broilers}\;\text{treated}\;\times \;\text{estimated}\;\text{chicken}\;\text{weight}\;\text{on}\;\text{treatment}\;\text{day}\;\left(\text{kg}\right)}$$

In these equations, farm-specific average daily body weights (kg) were estimated using a generalized additive model (‘gam’ function from *mgcv* package, version 1.9-3) (Wood, 2000, 2011), based on weight measurements of three randomly selected chickens taken weekly at each farm.

***Milligrams of AMDs Used per Population Correction Unit (mg/PCU).*** The total amount of all AMDs (active moiety) used per batch, in milligrams, was divided by the population correction unit (PCU), yielding mg/PCU.

(3) $$\frac{\text{mg}}{\text{PCU}}=\;\frac{\text{Amount}\;\text{of}\;\text{AMDs}\;\text{used}\;\text{per}\;\text{monitored}\;\text{batch}\;\left(\text{mg}\right)}{\text{PCU}\;\left(\text{kg}\right)}$$

Where the numerator was computed as the amount of AMDs (active moiety) used in all treatment days in a batch based on adjusted UDD values:

(4) $$\sum_{d=1}^{D}\text{UD}D_{\text{adjust}}\times \;\text{number}\;\text{of}\;\text{broilers}\;\text{treated}\;\times \;\text{estimated}\;\text{chicken}\;\text{weight}\;\text{on}\;\text{treatment}\;\text{day}$$

where day d= 1, ..., D represents any days with treatments.

And the denominator, Population Correction Unit (PCU, was calculated by multiplying the total number of broilers in a monitored batch by a standardized theoretical weight of 1 kg for a broiler chicken at treatment, as defined by the European Surveillance of Veterinary Antimicrobial Consumption (ESVAC) (European Surveillance of Veterinary Antimicrobial Consumption (ESVAC), 2021). One PCU is therefore equivalent to 1 kg of broiler chicken.

***Treatment incidence (TI) based on UDD and DDD.*** AMU was also quantified using treatment incidence (TI) at the farm level as described by (Persoons et al., 2012). The Used Daily Dose (UDD) and Defined Daily Dose (DDD) were considered as parameters, resulting in two different formulas, where $TI_{\text{UDD}}$ and $TI_{\text{DDD}}$ describe the number of UDD or DDD administered per 100 broiler-days at risk, or, in other words, the number of chickens per 100 that are treated daily with one DDD or UDD.

(5) $$TI_{\text{UDD}}=\;\frac{\text{Amount}\;\text{of}\;\text{AMD}\;\text{used}\;\text{per}\;\text{monitored}\;\text{batch}\;\left(\text{mg}\right)}{\text{UD}D_{median}\;\left(\frac{\text{mg}}{\text{kg}}\right)\times \;\text{numberof}\;\text{days}\;\text{at}\;\text{risk}\;\times \;\text{chicken}\;\text{biomass}\;\text{at}\;\text{risk}\left(\text{kg}\right)\;}\;\times \;100\;\text{broiler}\;\text{at}\;\text{risk}$$

(6) $$TI_{\text{DDD}}=\;\frac{\text{Amount}\;\text{of}\;\text{AMD}\;\text{used}\;\text{per}\;\text{monitored}\;\text{batch}\;\left(\text{mg}\right)}{\text{DDD}\;\left(\frac{\text{mg}}{\text{kg}}\right)\times \;\text{numberof}\;\text{days}\;\text{at}\;\text{risk}\;\times \;\text{chicken}\;\text{biomass}\;\text{at}\;\text{risk}\left(\text{kg}\right)\;}\;\times \;100\;\text{broiler}\;\text{at}\;\text{risk}$$

In these equations, the numerator was calculated per drug, representing the amount of each AMD (active moiety) administered during a production cycle, calculated by summing the amounts administered across all treatment days using adjusted UDD values. Since a given AMD could be administered multiple times at different dosages within a batch, UDD*_median_* was the median of the UDD values computed for that AMD in that batch (Timmerman et al., 2006). For DDD, we used standard dosages from the European Surveillance of Veterinary Antimicrobial Consumption (ESVAC), which provides recommended daily dosages for most AMDs registered for broilers (European Medicines Agency (EMA), 2016). The ‘number of days at risk’ corresponded to the duration of the production cycle. The ‘chicken biomass at risk’ was calculated as the number of chickens in the flock at the beginning of production multiplied by their mean body weight. This mean body weight was estimated at 0.8 kg across batches, by dividing the summed total weight of chickens on treatment days by the corresponding number of treated chickens and the number of treatment days (Persoons et al., 2012).

### Generalized Additive Mixed Models for the Analysis of the Association Between AMU and Key Explanatory Variables

We hypothesize that AMU is shaped by age-dependent management practices and disease-related pressures, as reflected in mortality. To explore non-linear age-related patterns in AMU and to assess whether daily within-flock mortality explains additional variation once broiler age (in days) is accounted for, we fitted three logistic GAMMs. The binary response variable represented daily farm-level AMU status. Separate models were fitted using datasets corresponding to AMU for preventive, therapeutic, or any use. Then, cutpoints identified using GAMM analyses were used to describe time points when AMU trends change and to further divide the production cycle into several periods (phases), each with its distinct AMU trend. This subsequently justified the use of a two-state semi-Markov process (SMP) model to simulate AMU as a dynamic process of treatment initiation and duration across these distinct phases. Further details of the GAMM construction and cutpoints estimation are provided in Text S1.

### Bayesian Semi-Markov Process Model for Temporal AMU Assessment

***Semi-Markov Process Specification.*** We hypothesize that AMU throughout the production cycle is primarily driven by the initiation of preventive interventions rather than therapeutic treatments, and that these processes may vary across production stages. To test this hypothesis and identify critical age windows where antimicrobial stewardship should focus on reducing unnecessary prophylaxis, we modeled treatment status as a discrete-time, two-state semi-Markov process (SMP) within a Bayesian framework. This approach allowed us to distinguish the probability of treatment initiation and the duration of treatment events across distinct production phases defined by cutpoints. Posterior samples from the fitted model were then used to simulate AMU trajectories as a dynamic process of treatment initiation and duration.

Let $s_{f,d}$ denote the treatment status of farm f (f = 1, ..., N = 60) on day d of the production cycle (d = 0, ..., $d_{max}=161$), taking values in the state space S={0,1}, where 0 (state 0) denotes "no treatment" and 1 (state 1) denotes "under treatment".

For a farm f, daily treatment initiation was modeled only on days when the farm was not already under treatment on the previous day (i.e. $s_{f,d-1}=0$). Conditional on this, $s_{f,d}$ follows a Bernoulli distribution:

(7) $$s_{f,d}|s_{f,d-1}\;∼\;\text{Bernoulli}\left(p_{f,d}\right),$$

with $p_{f,d}$ the probability of initiating treatment on day d, formulated as a logistic regression

(8) $$\text{logit}\left(p_{f,d}\right)=\;\beta_{0}+\beta_{1}.d+\sum_{r=1}^{R}\beta_{C_{r}}{\left(d-C_{r}\right)}^{+}+\mu_{f}$$

Where $C_{r}$ (*r* = 1, 2, 3) are pre-defined cutpoints indicating abrupt changes in AMU trends during the production cycle, allowing the transition probability to exhibit different slopes at specified time intervals. ${\left(d-C_{r}\right)}^{+}=max\left(0,d-C_{r}\right)$ is a truncated term representing the time elapsed since the cutpoint $C_{r}$. $\beta_{0}$ is the intercept, $\beta_{1}$ captures the baseline temporal trend in AMU, while $\beta_{C_{r}}$ quantifies changes in slope after each cutpoint. $\mu_{f}∼N\left(0,\sigma_{\mu }^{2}\right)$ is a farm-level random effect to account for unobserved heterogeneity across farms. The province location of farms was considered during model development but was not retained in the final formulation, as its inclusion did not improve model fit, and the marginal posterior distribution of its coefficient was centered around zero.

For a farm *f* in state 1, the duration of the *k^th^* treatment was modeled as:

(9) $$\text{Duratio}n_{f,k}\;∼\;\text{Poisso}n^{+}\;\left(\lambda_{f,k}\right)$$

Where $\text{Poisso}n^{+}$ denotes the Poisson distribution truncated at 1. The mean treatment duration $\lambda_{f,k}$ was modeled as:

(10) $$\log\left(\lambda_{k}\right)=\alpha_{0}+v_{f}$$

With $\alpha_{0}$ the general intercept and $v_{f}∼N\left(0,\sigma_{v}^{2}\right)$ a farm-level random effect.

***Prior Distributions, Bayesian Model Implementation.*** Weakly informative priors were specified for all model parameters: *Normal*(0, 4) priors were assigned to all fixed regression coefficients, and an *InverseGamma*(1, 1) prior was specified for the variance of the farm-level random intercepts.

All models were fitted using Markov Chain Monte Carlo simulation (MCMC) in JAGS (Just Another Gibbs Sampler) (Plummer, 2022) and R 4.4.3 (R Core Team 2025). Four parallel MCMC chains were run for each model. After discarding 5,000 burn-in iterations, each chain generated 20,000 iterations, with a thinning interval of 2 applied to reduce autocorrelation. This yielded 10,000 posterior samples per chain, for a total of 40,000 per model. Convergence was assessed using multiple diagnostics. All parameters achieved effective sample sizes (ESS) greater than 1,000, which is sufficient for stable parameter estimation (Makowski et al., 2024). Gelman-Rubin statistics (Rhat) were all below 1.01, indicating satisfactory convergence (Plummer, 2017). Visual inspection of the trace plots indicates good chain mixing and stationarity. Pairwise plots of posterior distributions were also examined to assess parameter correlations and marginal distributions. Posterior predictive checks (PPCs) were conducted to evaluate model adequacy by comparing observed data patterns with model-generated predictions.

Separate Bayesian semi-Markov process models were fitted to two time series describing daily preventive and therapeutic AMU, considering all 60 monitored batches; these models are hereafter referred to as the P-AMU and T-AMU models, respectively. Posterior predictive AMU patterns, regardless of purpose, were derived by jointly simulating posterior predictive preventive and therapeutic AMU trajectories from the fitted P-AMU and T-AMU models and aggregating their outputs at the farm- and day-level.

Median posterior predictive daily number of farms with preventive, therapeutic, and any AMU were computed, along with their 95% high-density intervals (HDIs). Further sensitivity analyses by shifting both cutpoints by 1, 2, and 3 days (Scenarios S1–S3, respectively) were conducted, with the simultaneous 95% CI of the temporal derivative for these shifted cutpoints including zero. The Bayesian SMP model was refitted under each scenario, with all other model specifications held constant. Phase-specific slopes were then compared across the three sensitivity scenarios. Predicted daily probabilities at the respective peak days within each phase were also compared across scenarios for preventive and therapeutic AMU. All predictions were generated based on a typical farm, with the farm-level random intercept set to zero.

## Results

### Descriptive results

***Study Batch Cycles.*** The median duration of production cycles across the monitored batches from 60 farms was 129 days (interquartile range, IQR: 117-146 days), with one notable exception (Farm 4) having a shorter cycle of 31 days. Each batch contained a median of 1,500 broilers (IQR: 1,000–1,500). Observed daily mortality risk patterns are shown in Figure S1, with the highest population-level of daily risk values observed during the first few days after hatching, reaching about 3‰ on day one after placement.

***AMD Treatments.*** A total of antimicrobial-substance 569 treatment events were recorded, with a median of 8 events per cycle (IQR: 6-11 events/cycle). Treatment duration ranged from one to eight days per event. Twenty-nine AMDs from 11 antimicrobial classes were identified. The most frequently administered AMDs were colistin (n = 107 events, 18.8%), amoxicillin (n = 100, 17.6%), doxycycline (n = 94, 16.5%), florfenicol (n = 64, 11.2%), and enrofloxacin (n = 30, 5.3%) (Fig. 3A). Together with sulfamonomethoxine and ampicillin, these 7 AMDs accounted for 79.3% of all treatment events. These AMDs were used throughout the production cycle across farms, except for enrofloxacin, which was mostly administered between days 0 and 50 (Figure S2). Norfloxacin, listed in group B (restrict) according to the AMEG category, was used less frequently (n = 2, 0.4%). At the batch level, a median of 5 (IQR: 3-6) AMDs, up to 12, and 5 (IQR: 3-6) classes, up to 9, were used per production cycle. Most treatment events were administered for preventive purposes (n = 401, 70.5%), while the remainder were therapeutic (n = 168, 29.5%) (Table 1). The most frequently administered AMDs for preventive treatment were colistin (n = 93, 23.2%), amoxicillin (n = 77, 19.2%), and doxycycline (n = 47, 11.7%) (Fig. 3B), whereas those for treatment were doxycycline (n = 45, 26.8%), florfenicol (n = 30, 17.9%), and amoxicillin (n = 23, 13.7%) (Fig. 3C). Respiratory (n = 97, 16.9%) and enteric diseases (n = 71, 12.6%) were the indications for treatment (Table 1). Notably, doxycycline (n = 38), florfenicol (n = 24), and tilmicosin (n = 9) were the predominant choices for treating respiratory conditions with clinical signs of gasping and difficulty breathing. In contrast, sulfamonomethoxine (n = 8) and trimethoprim (n = 6) were commonly used for enteric cases presenting with a blackish discoloration of the head.

**Fig. 3:**
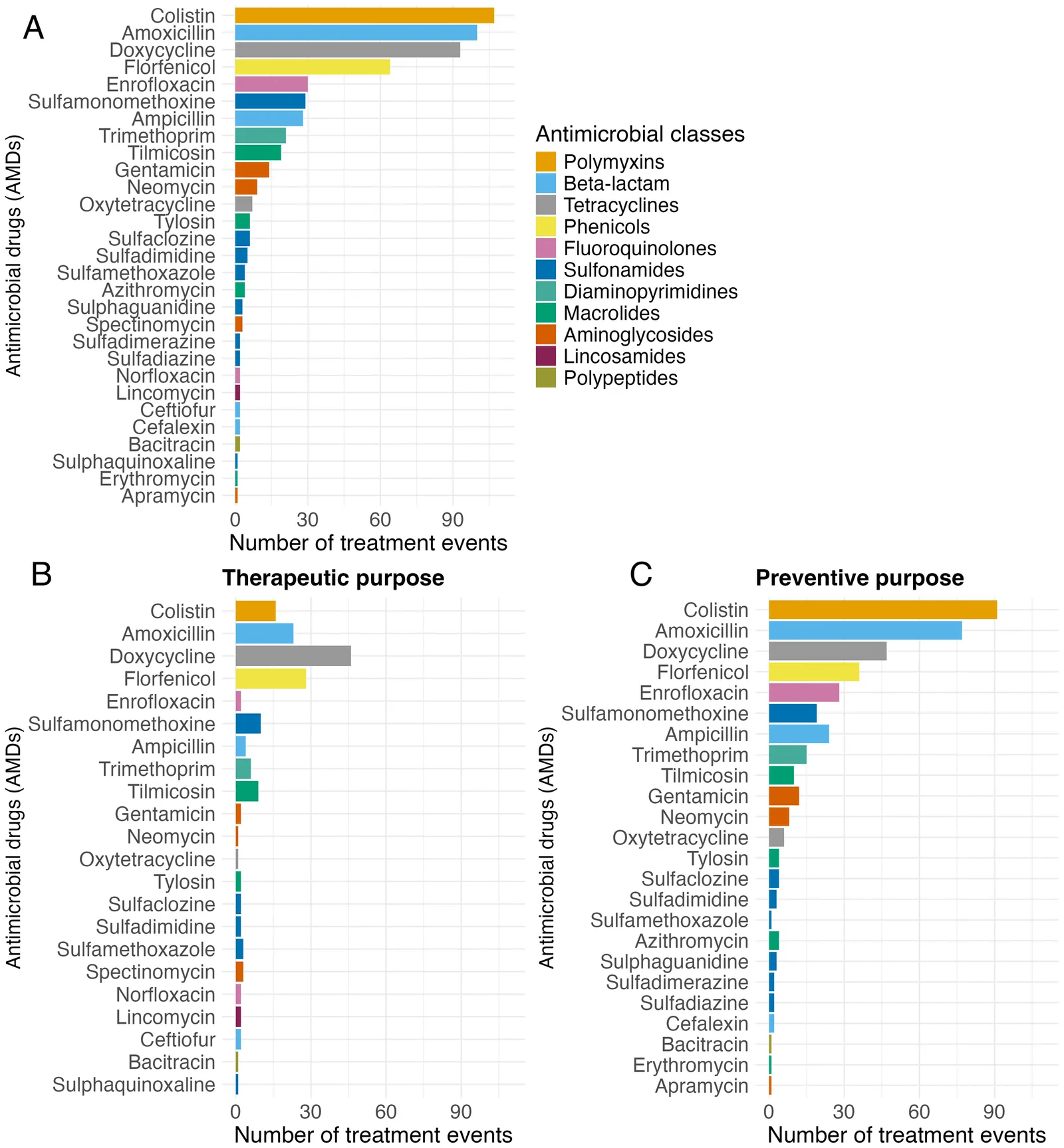
(A–C). Number of antimicrobial treatment events recorded over the production cycle in monitored batches from 60 farms, stratified by individual AMD and their respective classes, with A for any purpose, B for therapeutic, and C for preventive purposes.

**Table 1:** The number and percentage of treatment events based on therapeutic and preventive purposes, as well as clinical signs for the former.

Category	Clinical signs	No. of events (n)	Percentage (%)
Therapeutic usage	Respiratory	97	16.9
	• Gasping, difficulty breathing	91	16
	• Swollen eyes	2	0.3
	• Swollen comb	2	0.3
	• Nasal discharge	2	0.3
	Enteric	71	12.6
	• Bloody/mucoid droppings (may indicate coccidiosis)	5	0.9
	• Bloody droppings	10	1.8
	• White/chalky droppings	1	0.2
	• Diarrhea/loose droppings	37	6.5
	• Sulfur-yellow droppings, with a blackish appearance of the skin of the head (may indicate blackhead disease)	18	3.2
Preventive usage	-	401	70.5

***Temporal Trend of Daily AMU.*** The daily proportion of farms reporting AMU for preventive intervention (n=34, 56.7%) peaked early in the production cycle and hit the highest value on day two (Fig. 4A). However, the proportion of farms reporting AMU for therapeutic treatments increased during the later stages of the cycle and remained relatively stable despite slight fluctuations with a plateau at 11.9%-18.3% (n=7–11) from approximately day 19 to day 85 (Fig. 4B).

**Fig. 4:**
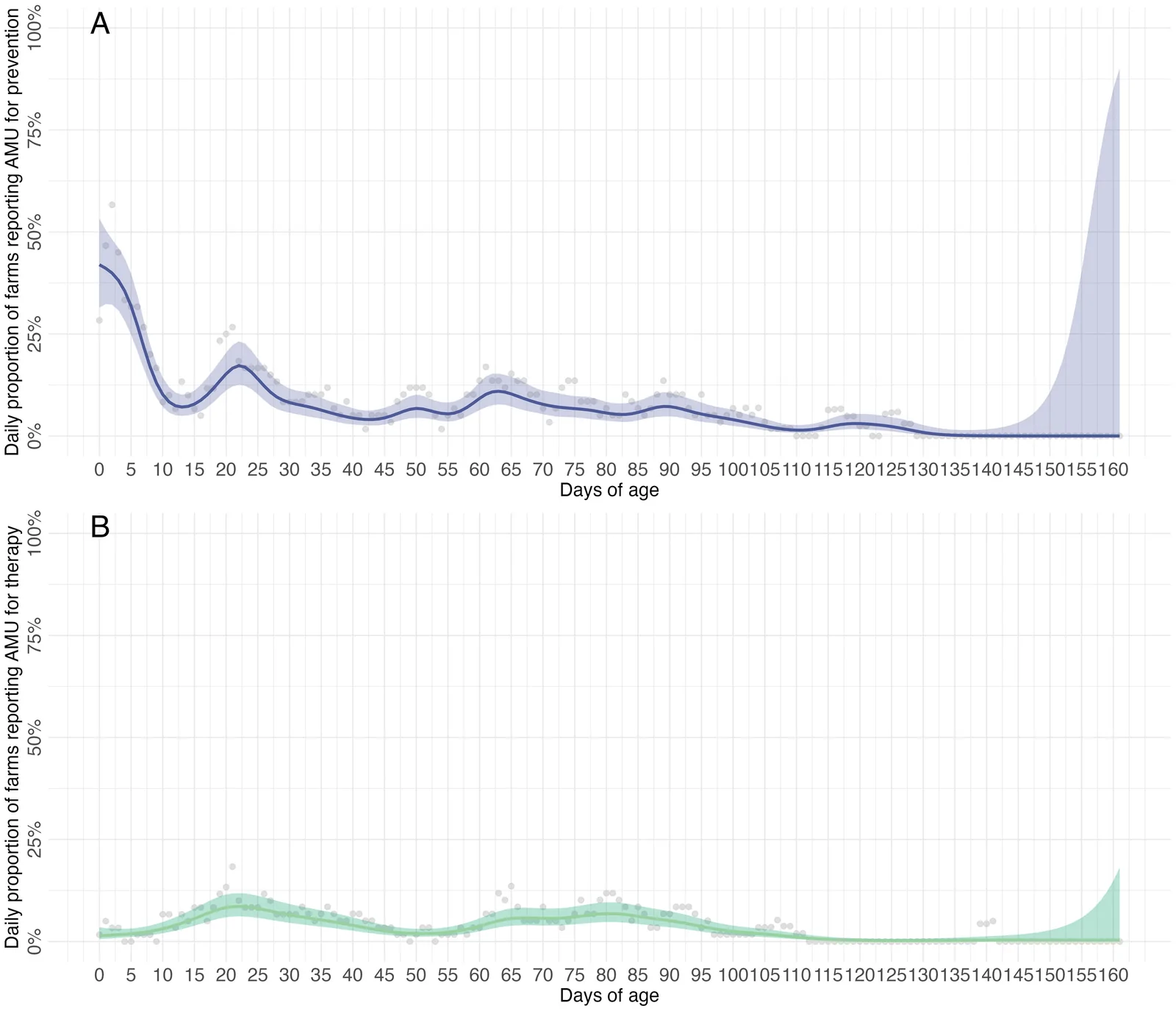
(A–B). Temporal trends in the daily proportion of farms reporting AMU for preventive interventions (A) and therapeutic treatments (B). The GAMM curve shows changes over time, with the shaded area on the plot reflecting the 95% CI. The widening of the 95% CI after day 150 is likely due to a reduced sample size, as the median production cycle across the 60 batches was 129 days (IQR: 117–146).

### AMU Quantification

Overall, 33.4% of the drug products contained more than one AMD, whereas 66.6% contained a single AMD. Most products were administered through drinking water (94%), with the remainder administered through feed. Regarding the actual daily dosage used on the farm, described as the UDD, it is presented in Table 2. The median UDD ranged from 2.3 mg/kg for colistin to 114.1 mg/kg for apramycin. However, some apparent high calculated UDD values (> 150 mg/kg/d) were reported on certain farms on specific days for the preventive or therapeutic use of amoxicillin, florfenicol, doxycycline, and sulfamonomethoxine used combined with trimethoprim (sulfamonomethoxine_TMP), with detailed information provided in Table S2. Among the most frequently used AMDs, higher doses early in the cycle (weeks 1-4) were observed for amoxicillin and florfenicol, for both preventive and therapeutic use (Figure S3). During the same period, higher therapeutic doses were also observed for colistin, doxycycline, and sulfamethoxazole-TMP.

**Table 2:** Summary of the adjusted used daily dose (UDD, mg/kg/d), $TI_{\text{UDD}}$ (number of broilers per 100 treated with one UDD), and $TI_{\text{DDD}}$ (number of broilers per 100 treated with one DDD) per AMD. AMDs were listed based on different priority AMEG categorization defined by EMA, where “category B-restrict”, category C-caution”, and “category D-prudence”.

Antimicrobial	Category	DDD	UDD median, (min-max)	TI_UDD median, (min-max)	TI_DDD median, (min-max)
Colistin	B	5.1	2.3 (0.1-257.2)	2.1 (0.0-207.6)	4.5 (0.1-438.4)
Enrofloxacin	B	10	23.5 (2.6-106.9)	0.2 (0.1-2.0)	0.7 (0.2-6.0)
Norfloxacin	B	-	47.4 (46.6-48.3)	1.4 (1.4-1.4)	-
Florfenicol	C	30	23.4 (0.4-277.0)	1.0 (0.1-33.5)	1.1 (0.1-37.2)
Tilmicosin	C	18	21.0 (11.1-46.8)	4.2 (0.8-14.1)	5.7 (1.1-19.2)
Tylosin	C	81	11.8 (6.5-149.6)	0.6 (0.4-1.4)	0.2 (0.1-0.6)
Neomycin	C	24	9.2 (1.3-24.8)	0.7 (0.2-8.0)	0.3 (0.1-3.3)
Gentamicin	C	-	13.4 (0.4-57.8)	1.4 (0.1-5.9)	-
Cefalexin	C	-	28.4 (26.4-68.4)	5.0 (4.2-5.8)	-
Apramycin	C	81	114.1 (103.8-125.4)	0.4 (0.4-0.4)	0.5 (0.5-0.5)
Azithromycin	C	-	20.6 (17.1-26.6)	3.7 (3.7-3.7)	-
Ampicillin	D	108	11.1 (1.3-69.2)	1.9 (0.1-17.6)	0.3 (0.0-2.3)
Amoxicillin	D	16	29.7 (2.7-417.1)	2.8 (0.1-14.1)	9.2 (0.5-46.8)
Doxycycline	D	15	22.4 (0.2-243.2)	3.2 (0.2-35.3)	6.2 (0.4-68.5)
Sulfamonomethoxine (alone)	D	9.5	45.1 (18.5-112.4)	1.7 (1.0-3.6)	10.7 (6.2-23.1)
Sulfamonomethoxine (combination with trimethoprim)⁎	D	-	32.8 (6.2-195.3)	2.6 (0.2-12.9)	-
Sulfaclozine	D	70	20.9 (5.6-79.5)	2.3 (1.2-3.0)	0.9 (0.5-1.2)
Sulfadimidine	D	182	19.4 (5.1-31.6)	3.2 (0.7-5.6)	0.3 (0.1-0.6)
Trimethoprim	D	6.4	7.1 (0.4-52.5)	2.0 (0.0-15.2)	2.8 (0.0-21.6)
Sulfadiazine	D	34	22.6 (6.4-24.3)	1.9 (1.5-2.2)	0.9 (0.8-1.1)
Sulfamethoxazole	D	27	28.8 (16.9-35.8)	1.9 (1.4-8.4)	2.0 (1.5-8.9)
Oxytetracycline	D	39	22.9 (6.8-100.0)	0.3 (0.1-10.2)	0.3 (0.0-8.2)
Sulfadimerazine	D	-	52.4 (47.4-57.4)	0.6 (0.6-0.6)	-
Sulphaquinoxaline	D	-	54.8 (51.5-58.5)	0.8 (0.8-0.8)	-
Sulphaguanidine	D	-	4.5 (3.4-12.8)	1.0 (1.0-1.0)	-
Bacitracin	D	-	34.2 (24.7-35.9)	4.4 (3.5-5.3)	-

⁎ : Combination products of sulfonamides with trimethoprim. ‘-’ For certain AMDs, TIDDD could not be computed because a standardized DDDvet value was not available from the European Surveillance of Veterinary Antimicrobial Consumption (ESVAC) (European Medicines Agency (EMA), 2016).

At the farm level, the overall antimicrobial consumption under treatment showed the median AMU to be 186.1 mg/PCU per cycle for preventive use (IQR: 23.7-411.7; mean ± SD: 349.7 ± 505.9), with a maximum of 2,973.3 mg/PCU, and 116.7 mg/PCU per cycle for therapeutic use (IQR: 10-237.8; mean ± SD: 207.8 ± 458.8), with a maximum of 3,432.5 mg/PCU.

For treatment frequency based on actual administered doses (quantified as $TI_{\text{UDD}}$), the median $TI_{\text{UDD}}$ for all drugs per monitored batch was 11.5 (IQR: 1.5-31.6) for AMDs used prophylactically and 6.4 (IQR: 3.1-15.4) for AMDs used therapeutically. This indicates that, on a given day, 11.5 and 6.4 chickens per 100 chickens were treated with one UDD for preventive and therapeutic purposes, respectively. When considering per AMD, $TI_{\text{UDD}}$ and $TI_{\text{DDD}}$ for each AMD are shown in Table 2. A higher $TI_{\text{DDD}}$ relative to $TI_{\text{UDD}}$ (Δ≥1) was observed for certain AMDs, including colistin (median $TI_{\text{DDD}}$: 4.5 vs. $TI_{\text{UDD}}$: 2.1), tilmicosin (median $TI_{\text{DDD}}$: 5.7 vs. $TI_{\text{UDD}}$: 4.2), amoxicillin (median $TI_{\text{DDD}}$: 9.2 vs. $TI_{\text{UDD}}$: 2.8), doxycycline (median $TI_{\text{DDD}}$: 6.2 vs. $TI_{\text{UDD}}$: 3.2), sulfamonomethoxine (median $TI_{\text{DDD}}$: 10.7 vs. $TI_{\text{UDD}}$: 1.7), where fewer chickens were being treated than expected based on the DDD.

### Association between AMU, Broiler Age, and Within-flock Mortality

Broiler age was strongly associated with the probability of a farm having AMU, for all/any purpose (*p* < 0.001), after accounting for within-flock mortality and the interaction between mortality and broiler age (Table 3). However, within-flock mortality was not associated with the probability of a farm reporting preventive AMU (*p* = 0.9), therapeutic AMU (*p* = 0.6), or AMU regardless of purpose (*p* = 0.2) after adjusting for broiler age and the age-mortality interaction (Table 3). Partial effect plots (on the logit scale) (Figure S4) show the individual effects of broiler age and within-flock mortality and their interaction. No association was found between within-flock mortality and any AMU outcome, as the 95% CI of the mortality-related smooth terms included 0 in all three models. Although model estimates suggested age-related variation in the associations between within-flock mortality risk and preventive (*p* = 0.01) and therapeutic AMU (*p* = 0.03) (Table 3), there was high uncertainty in the estimates, particularly at older ages and higher mortality levels where the partial plot showed relatively sparse observations and the 95% CI around the predicted three-dimensional surface widened (Figures S4-6).

**Table 3:** Summary of GAMM results, with additive effects of within-flock mortality, broiler age, and their interaction, and the random intercept of the farm.

Preventive AMU	Parameters	Estimate	SE	p-value
	• intercept	-2.82	0.15	<10^-15^
	Smoothing functions	Edf⁎	Ref.df⁎	p-value
	• age of broiler	5.67	6.67	<10^-15^
	• within-flock mortality	2.5	3.1	0.92
	• age of broiler × within flock mortality	2.26	2.65	0.01
	• farm	51.51	59	<10^-15^
Therapeutic AMU	Parameters	Estimate	SE	p-value
	• intercept	-3.6	0.16	<10^-15^
	Smoothing functions	edf	Ref.df	p-value
	• age of broiler	7.17	7.97	<10^-15^
	• within-flock mortality	2.02	2.51	0.61
	• age of broiler × within flock mortality	5.12	6.49	0.03
	• farm	45.42	59	<10^-15^
AMU regardless of purpose	Parameters	Estimate	SE	p-value
	• intercept	-2.13	0.09	<10^-15^
	Smoothing functions	edf	Ref.df	p-value
	• age of broiler	6.59	7.46	<10^-15^
	• within-flock mortality	2.94	3.65	0.25
	• age of broiler × within flock mortality	5.36	6.81	0.04
	• farm	48.26	59	<10^-15^

⁎ EDF = effective degree of freedom, which measures the actual model complexity (wiggliness) of a smooth term. A larger EDF implies more complex, wiggly splines (Wood, 2017). The EDF equals 1 if the smooth term is penalized to a simple linear relationship, while higher values indicate non-linear, more complex curves. Ref. DF = reference number of degrees of freedom used in computing test statistic and the p-values.

Although sensitivity analysis suggested a correlation between within-flock mortality and therapeutic AMU at all three lag structures (*p* = 0.01–0.04) and for any AMU when mortality was averaged over the preceding 2 and 3 days (*p* = 0.02 and 0.01), the estimated partial effects were close to zero in the low-mortality range for all cases, and their 95% CI included zero, showing high uncertainty (Table S3, Figure S7-9).

***Cutpoints.*** The predicted probability of a farm reporting AMU varied throughout the cycle, with cutpoints indicating the transition time points when temporal AMU trends shifted between increasing and decreasing phases, thereby partitioning the cycle into distinct AMU phases (Figure S10). For preventive AMU, cutpoints were identified at days 57 and 79. For therapeutic AMU, cutpoints occurred at days 27, 53, and 83. Cutpoints occurring beyond 125 days of age were not considered because uncertainty increased substantially during this period, with the derivative CI including zero throughout, likely reflecting the decreasing number of flocks as they were progressively sold (Simpson, 2018).

### Bayesian SMP Model Results for Temporal AMU Trends

***Convergence Check and Posterior Predictive Check.*** Convergence was achieved for both P-AMU and T-AMU models. Trace plots from the four chains showed good mixing and no visual indication of non-convergence (Figures S11-12). Pairwise plots of posterior samples (Figures S13-14) exhibited approximately elliptical ('Gaussian blob') patterns for most parameter pairs, indicating well-behaved posterior distributions with no strong non-linear collinearity.

PPCs for the P-AMU model revealed slight discrepancies between observed and predicted daily numbers of farms reporting AMU, with observed values occasionally falling outside the 95% highest density interval (HDI) (Figure S15). Specifically, a large underestimation was observed during the first four days of the cycle. For the T-AMU model, all but four observations, spanning days 60-65 and day 21, fell within the 95% HDI of the posterior predictive distribution. When considering any AMU purpose, the observed values fell outside the 95% HDI, mainly occurring on days 0-4, 18-20, and 60-66.

***Posterior Predictive Outputs in P-AMU and T-AMU Models.*** The identified cutpoints divided the production cycle into distinct AMU phases, each characterized by different temporal trends in the daily probability of initiating preventive or therapeutic treatment (Fig. 5B and D). At the start of a production cycle, initiation of preventive treatment was more likely than initiation of therapeutic treatment. On day 0, the estimated probability of initiating preventive AMU was 8.6 times that of initiating therapeutic AMU (RR = 8.6, 95% HDI: 3.4–17.7). During days 0–4, the daily observed proportion of farms receiving preventive AMU averaged 44.2% (range: 28.3–56.6%), compared with 3.3% for therapeutic AMU (range: 1.7–5.0%), indicating a predominance of preventive AMU at the beginning of the production cycle. The model-estimated preventive-to-therapeutic initiation RR remained greater than 2 through day 22 (Fig. 5A). However, this difference progressively narrowed as the production cycle advanced, with preventive and therapeutic treatment initiation reaching similar probabilities by day 83 (RR=1.05, 95% HDI: 0.49-1.82).

**Fig. 5:**
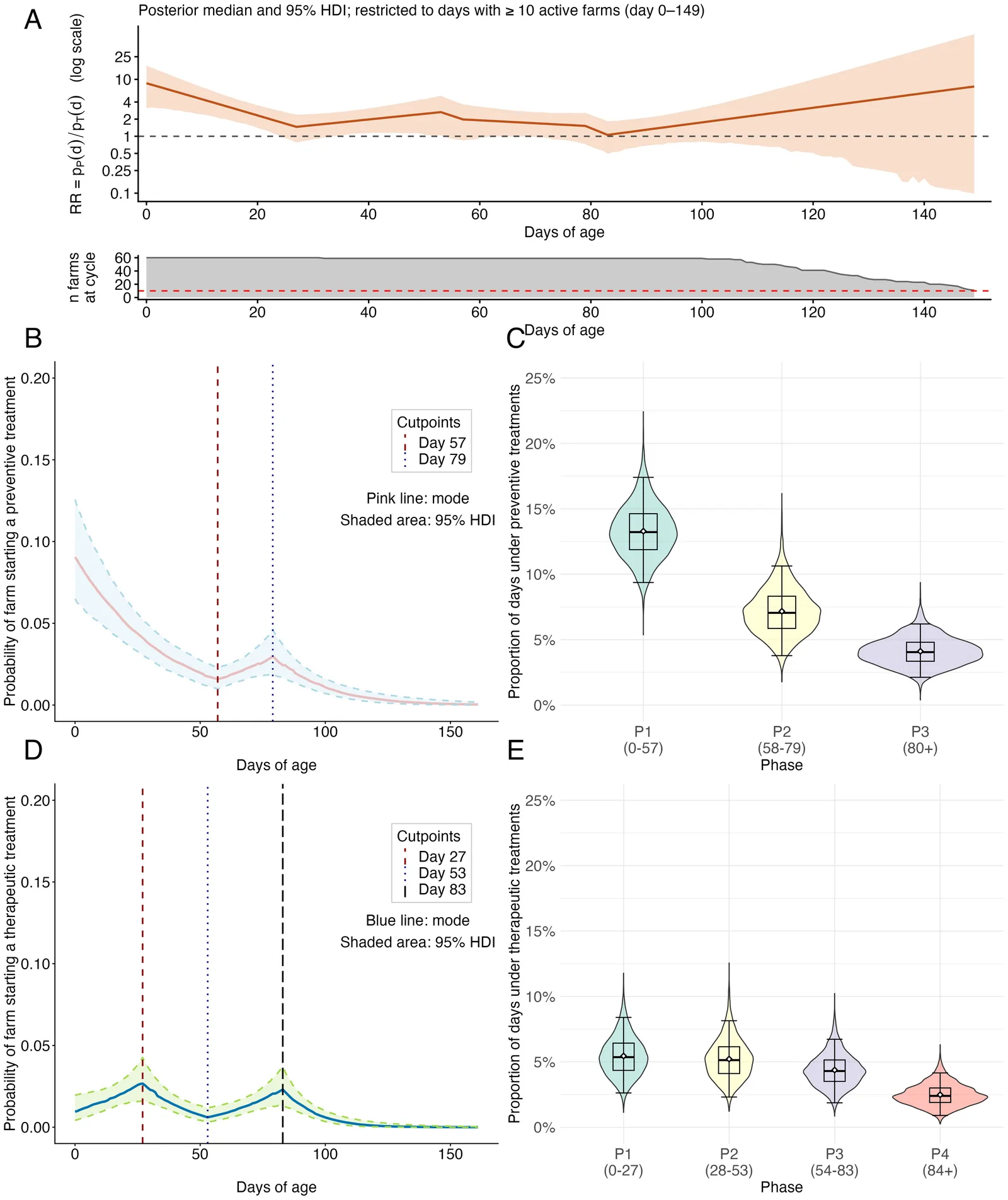
(A–E). In (A), the y-axis shows the risk ratio for initiating preventive vs. therapeutic treatment daily. The shaded area represents the 95% HDI, and the widening after day 120 is likely due to (1) the progressive decline in the number of farms still within their production cycle, where the median cycle length was 129 days (IQR 117–146), so fewer than half of the 60 batches contributed data beyond this point; (2) the instability of the ratio estimator when the denominator of the probability initiating a therapeutic treatment approaches zero at older ages. To avoid over-interpretation of these data-sparse regions, the curve was truncated at the last day on which ≥ 10 farms remained at risk. In (B-E), posterior quantiles of the probability of a farm starting preventive intervention (B) or therapeutic treatment (D) per cycle, and the predicted proportion of days under preventive (C) or therapeutic (E) treatments per phase. In (B) and (D), the horizontal line represents the median; the diamond indicates the mean; the box shows the IQR (25th–75th percentiles); and the whiskers indicate the 95% HDI.

This early dominance of prophylaxis was mainly driven by the higher probability of initiating preventive treatment during the first phase of production (days 0-57) compared to the second phase (days 58-79; RR=1.87, 95%HDI:1.39-2.39) and final phase (Day 80+; RR = 6.01, 95% HDI: 4.03-8.38) (Fig. 5B). The corresponding proportion of days under preventive treatment declined across phases, from 13.3% (95% HDI: 9.2–17.4%) in the first phase to 7.2% (95% HDI: 3.9–11%) in the second phase, and 4.2% (95% HDI: 2.1–6.9%) in the final phase (Fig. 5C).

Therapeutic treatment initiation was divided into four phases (days 0-27, 28-53, 54-83, after day 83) (Fig. 5D), but differences in initiation probability across the first three phases were limited. Compared with the first phase, the probability of initiating therapeutic treatment was similar in the second (RR=0.81, 95% HDI: 0.54-1.13) and third phases (RR=0.79, 95% HDI: 0.47-1.2). Consequently, the proportion of days under therapeutic treatment remained relatively stable across the first three phases, ranging from 5.4% (95% HDI: 2.6–8.5%) to 4.4% (95% HDI: 2.1–7%), before decreasing to 2.7% (95% HDI: 1.1–4.8%) in the final phase (Fig. 5E).

In the sensitivity analyses, the phase-specific slopes varied only marginally across scenarios for both the preventive and therapeutic AMU models (Table S4). Posterior median predicted probabilities at the phase-specific peak days also remained unchanged, except for preventive AMU at day 68 in Scenario S3, where the predicted probability was 0.03, compared with 0.02 in the original scenario (Table S5).

## Discussion

Our study quantified AMU on semi-intensive, slow-growing broiler farms in northern Vietnam over an entire production cycle. All 60 farms reported administering at least one AMD, resulting in 569 treatment events, most of which were preventive (70.5%). These findings were similar to a study conducted in Tien Giang Province during 2012–2013, in which only 59.1% of farms reported using antimicrobial formulations and 84% of administrations were for prevention (Carrique‐Mas et al., 2015). In both studies, preventive intervention remains the primary motivation for AMU. In this study we identified 29 AMDs from 11 antimicrobial classes. This is comparable to a study conducted in Thai Nguyen province, Vietnam, which focused on the final stage of the production cycle and identified 25 unique AMDs across 14 classes among 236 farms reporting AMU (Kemunto et al., 2026). The frequent use of colistin in our study (n = 107 events, 18.8%) and enrofloxacin (n = 30 events, 5.3%), primarily for preventive treatments, is concerning, as both are listed in AMEG Group B (Restrict), where colistin is important for treating severe infections caused by multidrug-resistant (MDR) bacteria in humans (Mahmood et al., 2025; Rojek et al., 2025). We also observed low-frequency use of two other Group B AMDs, ceftiofur and norfloxacin, with one treatment event recorded for each. Other frequently used AMDs for prevention included amoxicillin (Group D) and ampicillin (Group D), classified by AMEG to be used with 'prudence’ (European Medicines Agency (EMA), 2020) but also listed as 'critically important for human medicine' according to WHO recommendations (World Health Organization, 2024). In Vietnam, the preventive use of these high-priority AMDs, based on WHO criteria, has been officially banned for terrestrial animals since 2021, with a complete prohibition on all prophylactic use scheduled for 2026 (Carrique-Mas et al., 2023; Vietnamese Government, 2020). Drugs used for therapeutic treatment were mainly administered for respiratory and enteric diseases, which were given as the main reasons for their use. In particular, sulfamonomethoxine and trimethoprim were among the drugs most commonly used. When administered in combination, they constitute a potentiated sulfonamide regimen used in veterinary medicine to treat a variety of bacterial and protozoal infections, including coccidiosis in poultry (Stastny et al., 2023; World Organisation for Animal Health (OIE), 2021).

Amoxicillin is indicated for the treatment of susceptible infections in poultry that affect the alimentary, urogenital, and respiratory tracts, including pathogens associated with the chronic respiratory disease (CRD) complex and *E. coli* infections (Australian Pesticides and Veterinary Medicines Authority (APVMA), 2019). It has historically been used as a growth promoter but has been prohibited for this purpose in Vietnam since 2018 (Ministry of Agriculture and Rural Development, 2016). Sulfamonomethoxine and trimethoprim are used in combination as a potentiated sulfonamide in veterinary medicine to treat a variety of bacterial and protozoal infections, most notably coccidiosis in poultry (Stastny et al., 2023; World Organisation for Animal Health (OIE), 2021).

In our study, we used mg/PCU to measure overall antimicrobial consumption at the farm level, with a median AMU of 116.7 mg/PCU for therapy and 186.1 mg/PCU for prevention per production cycle. This is an order of magnitude lower than another study in northern Vietnam which has reported values of 2,167.1 mg/PCU in Bac Giang and 1,530.9 mg/PCU in Dong Thap (Ha et al., 2021). Nevertheless, the AMU consumption recorded in our study is still an order of magnitude higher than other global trends. For instance, the British Poultry Council reported a usage level of 11.33 mg/PCU for chickens in the United Kingdom (UK) in 2024 (British Poultry Council (BPC), 2025). In that same year, another European Union country (Iceland) reported that no antimicrobials were used in chickens (European Sales and Use of Antimicrobials for veterinary medicine (ESUAvet), 2024). Global estimates of AMU in chicken production showed a downward trend, from 148 mg/PCU in 2010 to approximately 35 mg/PCU in 2020, despite growth in the scale of production (Mulchandani et al., 2023).

For treatment frequency, the median $TI_{\text{UDD}}$ was 11.5 for prophylactic AMU and 6.4 for therapeutic AMU, corresponding to an average of 11.5 and 6.4 chickens per 100, respectively, receiving one UDD per day. A previous study conducted in Pakistan reported a median overall $TI_{\text{UDD}}$ of 75.3 (Mahmood et al., 2024), which targeted commercial broilers with shorter life cycles. However, our data revealed extremely high calculated UDD values (> 150 mg/kg/d) for amoxicillin, florfenicol, doxycycline, and apramycin. These values may suggest that farmers did not follow the dosages recommended on package labels or prescribed by veterinarians, possibly because of misunderstandings of label instructions, limited veterinary guidance, or errors in recording the amount of product used per treatment day or treatment course. Such overdosing may be a potential risk factor for the development of antimicrobial resistance. Efforts to control and optimize AMU should specifically target this early rearing phase.

Early-cycle preventive treatment was the main driver of AMU rather than therapeutic treatment. This may reflect farmers’ perception that young birds are more vulnerable at the start of the cycle with their use of AMD influenced by the need to prevent disease, mortality, and economic loss rather than by observation of clinical signs. The lack of association between within-flock mortality and farms’ daily AMU status can therefore be partly explained by the predominance of early preventive treatments, which were often initiated before observable increases in mortality. In contrast, during the mid-to-late stages of the production cycle, the differences in the likelihood of initiating preventive and therapeutic treatments narrowed. A similar increase in therapeutic use during this period was observed in traditional free-range broiler farms in France (Adam et al., 2019). This temporal pattern may reflect a shift from perceived vulnerability to observed disease pressure, when farmers are less inclined to undertake preventive measures and instead treat the flock as soon as they observe signs of disease or pathogen infestation, before the condition worsens. At the end of the production cycle, the likelihood of initiating treatment was low, regardless of purpose.

A similar trend was also reported in studies of small-scale slow-growing broiler flocks in the Mekong Delta region, where the probability of AMU decreased with flock age and was lowest at the end of the cycle (Cuong et al., 2021; Nhung et al., 2023). At this stage, broilers are typically ready for collection by traders. The lower probability of AMU use at this stage may therefore reflect regulatory considerations: AMU should be reduced to comply with withdrawal periods (WPs), defined as the minimum time required between the last administration of a veterinary drug and slaughter or food production to ensure that AMD residues in animal tissues (such as muscle) fall below the established maximum residue limit (MRL) (European Medicines Agency (EMA), 2025). Therefore, reducing AMU near slaughter may help lower the direct risk of antimicrobial residues entering the food chain, with WPs being respected.

This study has limitations, primarily related to self-reported AMU data. First, farmers recorded the AMDs used and the context of administration, which may have introduced reporting errors because of intentional underreporting or misclassification of purpose, or unintentional uncertainty about product identity, package instructions, or AMDs pre-mixed in feed. To minimize recall bias, the research team’s veterinarians contacted the farmers every three days to collect this information. Although reporting bias cannot be ruled out, the observed AMU patterns were plausible and were supported by this frequent, veterinarian-led follow-up. Future studies that combine AMU monitoring with residue detection would better capture actual drug administration.

For AMU quantification, we assumed that all antimicrobial intake from medicated feed was completely consumed. This assumption may overestimate the actual amount ingested by birds, as some feed may be left uneaten or wasted in field conditions. Meanwhile, TI was calculated using a standardized body weight at treatment across batches. This approach may not capture between-farm variation in body weight at treatment or within-cycle changes in body weight among slow-growing broilers.

For GAMM analysis, the temporal ordering between mortality and AMU might be uncertain, as mortality could occur after treatment had already been initiated. To address this potential temporal misalignment, sensitivity analyses using previous-day mortality and mean mortality over the preceding 2 and 3 days were conducted, which did not provide robust evidence that lagged mortality was associated with AMU. Nevertheless, since potential causal link between mortality levels observed by the farmer and their decision to treat could not be established from the available data, potential reverse causation cannot be completely excluded as well. Meanwhile, AMU was recorded daily within farms, and administration could continue over several consecutive days during a treatment event. This recording structure may introduce temporal dependence that the GAM models do not fully capture. However, assessing residual autocorrelation in our binomial GAMMs depends on the residual definition, model specification, and the data's temporal structure, including the binomial response distribution, the logit link, smooth and random-effect terms, and repeated daily observations within farms. For binary outcomes, deviance residuals reflect the response's discreteness and vary with fitted probabilities, which complicates interpretation of their autocorrelation function (ACF). In our study, the 'acf_resid()' function from the itsadug package (version 2.5) (Van Rij et al., 2015) was used to generate ACF plots, suggesting substantial positive residual autocorrelation at a one-day lag, with weaker correlations persisting at lags of two to three days. GAM CIs should be wider than they are due to the likely presence of autocorrelation.

For Bayesian SMP analysis, GAMM-derived production-phase cutpoints were treated as fixed in the Bayesian semi-Markov model, and their estimation uncertainty was not propagated into phase-specific estimates. Therefore, sensitivity analyses shifting both cutpoints by 1–3 days, using the same model structures, showed minimally affected phase-specific slopes for preventive and therapeutic AMU and left predicted phase-specific peak probabilities for a typical farm essentially unchanged. These results support the robustness of the findings to the tested cutpoint shifts. Meanwhile, the Bayesian SMP model underestimated the number of farms receiving preventive AMU during days 0–4, while the raw data showed that preventive AMU was far more prevalent than therapeutic AMU during the first few days of the production cycle, reinforcing the early cycle as a critical target for antimicrobial stewardship. Thus, the day-0 risk ratio between preventive and therapeutic initiation might be greater than estimated, which should be interpreted as a conservative estimate.

Finally, although farms were randomly selected within each study area, sampling was restricted to two locations: one in Ha Noi and one in Bac Ninh provinces. Farming practices may vary more widely across northern Vietnam, and the sampled farms may therefore not be representative of all slow-growing broiler farms in the region.

## Conclusion

Our study shows that AMU was driven mainly by the initiation and use of preventive treatments early in the production cycle, while therapeutic use persisted during later growth stages, primarily in response to respiratory and enteric disease symptoms. The frequent use of critically important AMDs for human medicine, specifically enrofloxacin and colistin, highlights a significant public health concern. Therefore, targeted antimicrobial stewardship is crucial during the early cycle to reduce non-essential prophylactic use, restrict the use of critically important AMDs, and promote lower-risk alternatives and non-antimicrobial disease prevention strategies.

## Author contribution statement

**Chen Xin** curated and analyzed the data, contributed to methodological and software development, validated the results, prepared the visualizations, and drafted the manuscript. **Trang Nguyen** and **Quoc Hung Nguyen** contributed to the investigation, data collection, and data curation. **Saira Butt** coordinated project activities, curated the data, and contributed to methodological and software development. **Huong Quynh Luu** contributed to the investigation and data collection. **Hoa Thi Thanh Pham** contributed to the study conception and methodological development and coordinated project activities. **Anne Conan** contributed to the study conception, methodological development, data curation, and project coordination. **Ngoc Pham Thi** contributed to the study conception and methodological development, secured funding, provided research resources, and coordinated project activities. **Priscilla F. Gerber** contributed to methodological and software development, supervised the research, and contributed to drafting the manuscript. **Dirk Pfeiffer** contributed to the study conception, secured funding, provided research resources, and coordinated project activities. **Damer Blake** contributed to methodological development, secured funding, and coordinated project activities. **Fiona Tomley** contributed to the study conception, secured funding, and coordinated project activities. **Beatriz Calvo-Urbano** curated and analyzed the data, contributed to methodological and software development, supervised the research, prepared the visualizations, and contributed to drafting the manuscript. **Guillaume Fournié** and **Ludovic Pelligand** contributed to the study conception, methodological and software development, data curation, and data analysis. They also secured funding, coordinated and supervised the research, validated the results, prepared the visualizations, and contributed to drafting the manuscript.

All authors critically reviewed the manuscript for important intellectual content and explicitly approved the final version to be published. Each author agrees to be personally and publicly accountable for all aspects of the work. All authors undertake to ensure that any questions concerning the accuracy or integrity of any part of the work are thoroughly investigated and resolved.

## Data Availability Statement

The datasets are provided as Supplementary Material 4 (“VN_Daily_farm_records.csv”) and Supplementary Material 5 (“VN_AMU_Quantification.csv”) and are also available in the GitHub repository at: https://github.com/Millaaa-12/AMU-reproducibility-package

## Code Availability Statement

The underlying code for the analysis is available on GitHub and can be accessed via this link: https://github.com/Millaaa-12/AMU-reproducibility-package

## Disclosures

The authors declare that they have no known competing financial interests or personal relationships that could have appeared to influence the work reported in this paper.

## Footnotes

Supplementary material associated with this article can be found, in the online version, at doi:10.1016/j.psj.2026.107918.
