# Supplementary material for: Longitudinal study assessing antimicrobial usage in broiler production cycles in northern Vietnam in 2022 to 2023
Source: Poult Sci. 2026 Oct 6;105(12):107918. doi: 10.1016/j.psj.2026.107918 (PMC13694736; doi:10.1016/j.psj.2026.107918)
Supplement: Supplementary file 1 [file mmc1.docx]

# Supplementary Information

- Supplementary figures

**Figure S1**. Observed daily mortality risk (%) throughout the production cycle.

**Figure S2**. The distribution of antimicrobial treatment events in the production cycle of monitored batches in 60 farms, by individual AMD and their respective antimicrobial class, where the x-axis represents the age of broilers when any treatment ended, and the y-axis represents the density of treatment events.

**Figure S3**. Heatmap of the weekly mean used daily dose (UDD) for the top 12 frequently used AMDs, stratified by administration purpose. The upper panel displays UDDs for therapeutic purposes, while the lower panel illustrates preventive usage across treatment weeks. UDD values are expressed in milligrams per kilogram of body weight per day (mg/kg). To minimize the influence of extreme outliers, the color scale was clipped to the 5th and 95th percentiles (2.5–73.7 mg/kg). Blank (white) cells indicate weeks with no recorded usage for that specific AMD.

**Figure S4**. The partial effects (on the link scale) of each individual factor (i.e. mortality (top left) and broiler age (top right)) and of the interaction of broiler age and mortality (bottom left) (excluding the contribution of the individual factors), considering different response variables, including preventive AMU (A), therapeutic AMU (B), and AMU regardless of purposes (C).

**Figure S5.** Interaction between broiler age and within-flock mortality risk on the probability of (A) preventive, (B) therapeutic and (C) overall AMU. Red areas are where the 95% CI lies entirely above zero. Blue areas are where it lies entirely below zero. Grey areas are where the confidence interval includes zero. Black lines are contours of the estimated partial effect. Grid points far from the observed data were excluded.

**Figure S6.** Three-dimensional graphics, considering all terms (within-flock mortality, age of broiler and the interaction of broiler age and mortality), showing the combined three surfaces (one at the predicted values minus 2 SE, one at the predicted values, and one at the predicted values plus 2 SE) for preventive AMU (A), therapeutic AMU (B), AMU regardless of purpose(C). The x-axis is the age of the broiler, measured in days, the z-axis is the daily within-flock % mortality, the vertical scale of the graph (y-axis) is the linear predictor, representing the logit of the probability of a farm on any given day with preventive AMU (A), therapeutic AMU (B), and AMU regardless of purpose (C).

**Figure S7**. Partial effects on the link scale of broiler age, 1-day-lagged mortality, and their interaction for preventive AMU (A), therapeutic AMU (B), and AMU regardless of purpose (C). The top-left and top-right panels show the partial effects of 1-day-lagged mortality and broiler age, respectively, whereas the bottom-left panel shows the partial effect of their interaction, excluding the main effects of the individual variables.

**Figure S8.** Partial effects on the link scale of broiler age, mean mortality over the preceding 2 days, and their interaction for preventive AMU (A), therapeutic AMU (B), and AMU regardless of purpose (C). The top-left and top-right panels show the partial effects of mean mortality over the preceding 2 days and broiler age, respectively, while the bottom-left panel shows the partial effect of their interaction, excluding the main effects of the individual variables.

**Figure S9.** Partial effects on the link scale of broiler age, mean mortality over the preceding 3 days, and their interaction for preventive AMU (A), therapeutic AMU (B), and AMU regardless of purpose (C). The top-left and top-right panels show the partial effects of mean mortality over the preceding 3 days and broiler age, respectively, while the bottom-left panel shows the partial effect of their interaction, excluding the main effects of the individual variables.

**Figure S10.** The first-order derivative results as a rate of change of broiler age measured in days throughout the entire production cycle, for preventive AMU (A), and therapeutic AMU (B).

**Figure S11.** **Trace plots with four chains for the P-AMU model**: This figure shows the trace plots (n chains = 4, n iterations = 20000) of the P-AMU model, which displayed tight, consistent horizontal bands, indicating no visual signs of non-convergence within the chains. The parameter beta0 is the overall intercept in logistic regression, while alpha0 is the intercept in the Poisson regression model. The fixed-effect coefficient beta_interval0 captures the baseline temporal trend in preventive AMU, while the other two coefficients, beta_interval1 and beta_interval2, quantify the overall differences in linear trends after versus before CP1 and CP2. The parameters logistic_farm_sigma and poisson_farm_sigma represent the variances of the random intercepts in both the logistic regression and Poisson regression models.

**Figure S12. Trace plots with four chains for the T-AMU model:** This figure shows the trace plots (n chains = 4, n iterations = 20000) of the T-AMU model, which displayed tight, consistent horizontal bands, indicating no visual signs of non-convergence within the chains. The parameter beta0 is the overall intercept in logistic regression, while alpha0 is the intercept in the Poisson regression model. The fixed-effect coefficient beta_interval0 captures the baseline temporal trend in therapeutic AMU, while the other three coefficients, beta_interval1, beta_interval2, and beta_interval3, quantify the overall differences in linear trends after versus before CP1, CP2, and CP3. The parameters logistic_farm_sigma and poisson_farm_sigma represent the variances of the random intercepts in both the logistic regression and Poisson regression models.

**Figure S13. Pairs plot with four chains for the P-AMU model:** This figure shows the pairs plot of the P-AMU model, which showed the distribution of each parameter’s posterior values and its interactions with other parameters. The parameter beta0 is the overall intercept in logistic regression, while alpha0 is the intercept in the Poisson regression model. The fixed-effect coefficient beta_interval0 captures the baseline temporal trend in preventive AMU, while the other two coefficients, beta_interval1 and beta_interval2, quantify the overall differences in linear trends after versus before CP1 and CP2. The parameters logistic_farm_sigma and poisson_farm_sigma represent the variances of the random intercepts in both the logistic regression and Poisson regression models.

**Figure S14. Pairs plot with four chains for the T-AMU model:** This figure shows the pairs plot of the T-AMU model, which showed the distribution of each parameter’s posterior values and its interactions with other parameters. The parameter beta0 is the overall intercept in logistic regression, while alpha0 is the intercept in the Poisson regression model. The fixed-effect coefficient beta_interval0 represents the general change in daily therapeutic AMU over time, while the other three coefficients, beta_interval1, beta_interval2, and beta_interval3, quantify the overall differences in linear trends after versus before CP1, CP2 and CP3. The parameters logistic_farm_sigma and poisson_farm_sigma represent the variances of the random intercepts in both the logistic regression and Poisson regression models.

**Figure S15. Posterior predictive checks of the daily proportion of farms with preventive AMU based on the P-AMU model (a), therapeutic AMU based on the T-AMU model (b), and any AMU purpose based on both the P-AMU and T-AMU models (c).** Points indicate the observed daily proportions of farms with preventive AMU, therapeutic AMU, and any AMU. On each day, the shaded region shows the IQR (25th–75th percentiles) of the posterior predictive estimate, and the whiskers indicate the 95% HDI, all derived from the posterior predictive distributions.
