# Supplementary material for: Longitudinal study assessing antimicrobial usage in broiler production cycles in northern Vietnam in 2022 to 2023
Source: Poult Sci. 2026 Oct 6;105(12):107918. doi: 10.1016/j.psj.2026.107918 (PMC13694736; doi:10.1016/j.psj.2026.107918)
Supplement: Supplementary file 2 [file mmc2.docx]

# Supplementary Information

**Text S1**

Here, we provided further information about the GAMM and the cutpoints calculation.

**Generalized additive mixed models for the analysis of the association between AMU and key explanatory variables**

To investigate the association between AMU and key explanatory variables, namely broiler age (in days), daily within-flock mortality (as a percentage), and their interaction (daily changes in mortality), we fitted three logistic generalized additive mixed models (GAMMs). The binary response variable represented daily farm-level AMU status. Separate models were fitted using datasets corresponding to AMU for preventive, therapeutic, or any use. Each model incorporated farm-specific daily within-flock mortality values and broiler age as explanatory variables. The models were expressed as:

$logit\left( p_{ij} \right)=\beta_{0\_g}+ f_{1}({day}_{ij})+ f_{2}\left( {mortality}_{ij} \right)+ f_{3} ({day}_{i}, {mortality}_{ij})+b_{j}$ (1)

Where $p_{ij}$ represents the probability of a farm $j$ reporting AMU on day $i$. The intercept $\beta_{0\_g}$ represented the log-odds of the baseline AMU probability when broiler age and mortality risk were zero. Non-linear effects of broiler age and daily within-flock mortality were modeled using functions $f_{1}({day}_{ij})$ and $f_{2}\left( {mortality}_{ij} \right)$, respectively. Their non-linear interaction was captured via a tensor-product bivariate smooth function $f_{3} ({day}_{ij}, {mortality}_{ij}$). A farm-level random effect $b_{j}$ was used to account for repeated measurements within farms and unobserved between-farm heterogeneity. Smooth terms were estimated as penalized regression splines, with smoothing parameters selected by restricted maximum likelihood (REML), using the 'gam' function from the *mgcv* package (version 1.9-3) (Wood, 2000). The 'k.check' function from the same package was used to check the adequacy of smooth basis dimension ($k$), where the combination of low p-values (<0.05) and effective degrees of freedom (edf) close to $k$ should be avoided. Two outliers were excluded prior to model fitting as they exceeded 1.5 times the interquartile range of daily within-flock mortality.

Partial effect plots were generated via the 'draw' function from the *gratia* package (version 0.11.1) (Simpson, 2019), showing the contributions of broiler age, daily within-flock mortality, and their interaction on the logit-scale with 95% confidence interval (CI), with all other model terms centered so that their contribution was zero. The interaction between broiler age and mortality was visualized using the 'visreg2d' function from the same package. Meanwhile, the three-dimensional surfaces of predicted surface and uncertainty bounds +/-2 standard errors on the logit scale) were generated using the 'gam' function from the *mgcv* package.

Sensitivity analyses were conducted to explore the impact of temporal lag on the correlation between within-flock mortality and AMU. Same-day mortality was replaced with previous-day mortality or mean mortality over the preceding 1, 2, and 3 days. The same model structure and observation set were retained across analyses.

**Definition of cutpoints**

Cutpoints (time points at which AMU trends changed) were identified using GAMM analyses and used to divide the production cycle into several periods (phases) with distinct AMU trends. This subsequently justified the use of an SMP model to simulate AMU as a dynamic process of treatment initiation and duration across these distinct phases.

Specifically, the marginal effect of broiler age on the probability of AMU at the farm level (response scale) was computed using the 'plot_slopes' function from the *marginaleffects* package (version 0.30.0) (Arel-Bundock, 2021). Marginal effects, defined as partial derivatives of the fitted regression function, measure the instantaneous rate of change in the outcome, the probability that a farm reports AMU, with respect to broiler age, holding other variables constant. Positive derivative values indicate an increasing probability of AMU with age, whereas negative values indicate a decreasing probability. Periods of significant temporal change were identified as time points at which simultaneous 95% CIs for the derivative exclude zero, indicating statistically significant changes in the response over time (Simpson, 2018). Therefore, cutpoints were the first time point following each directional shift in the derivative (i.e., from positive to negative value, or from negative to positive value) for which the 95% CI did not include zero.

Reference:

Arel-Bundock, V., 2021. marginaleffects: Predictions, comparisons, slopes, marginal means, and hypothesis tests. https://doi.org/10.32614/CRAN.package.marginaleffects

Simpson, G.L., 2019. gratia: Graceful ’ggplot’-based graphics and other functions for GAMs fitted using “mgcv.” https://doi.org/10.32614/CRAN.package.gratia

Simpson, G.L., 2018. Modelling palaeoecological time series using generalised additive models. Front. Ecol. Evol. 6, 149. https://doi.org/10.3389/fevo.2018.00149

Strang, G., Herman, E. “Jed,” n.d. Derivatives and the shape of a graph, in: Calculus. LibreTexts.

Wood, S., 2000. mgcv: Mixed GAM computation vehicle with automatic smoothness estimation. https://doi.org/10.32614/CRAN.package.mgcv
