# Supplementary material for: Longitudinal study assessing antimicrobial usage in broiler production cycles in northern Vietnam in 2022 to 2023
Source: Poult Sci. 2026 Oct 6;105(12):107918. doi: 10.1016/j.psj.2026.107918 (PMC13694736; doi:10.1016/j.psj.2026.107918)
Supplement: Supplementary file 3 [file mmc3.docx]

# Supplementary Information

**Table S1** Conversion of content in veterinary antimicrobial drugs (AMDs) into active moiety equivalents, where labelled masses of AMD derivatives were converted to the pharmacologically active moiety using molecular weights retrieved from PubChem (Kim et al., 2023)

| Active ingredient from veterinary drug products | Derivative molecular weight (g/mol) | AMD (Active moiety) | Active moiety molecular weight (g/mol) | Active moiety per mg of derivative |
| --- | --- | --- | --- | --- |
| Amoxicillin trihydrate | 419.5 | Amoxicillin | 365.4 | 0.87 |
| Cephalexin monohydrate | 365.4 | Cephalexin | 347.4 | 0.95 |
| Doxycycline hyclate | 545 | Doxycycline | 444.4 | 0.82 |
| Enrofloxacin hydrochloride | 395.9 | Enrofloxacin | 359.4 | 0.91 |
| Erythromycin thiocyanate | 793 | Erythromycin | 733.9 | 0.93 |
| Gentamicin sulfate | 575.7 | Gentamicin | 477.6 | 0.83 |
| Lincomycin hydrochloride | 443 | Lincomycin | 406.5 | 0.92 |
| Neomycin sulfate | 908.9 | Neomycin | 614.6 | 0.68 |
| Norfloxacin hydrochloride | 355.79 | Norfloxacin | 319.33 | 0.9 |
| Oxytetracycline hydrochloride | 496.9 | Oxytetracycline | 460.4 | 0.93 |
| Spectinomycin dihydrochloride | 405.3 | Spectinomycin | 332.35 | 0.82 |
| Spectinomycin hydrochloride | 368.81 | Spectinomycin | 332.35 | 0.9 |
| Spectinomycin sulphate | 430.43 | Spectinomycin | 332.35 | 0.77 |
| Sulfaclozine sodium | 306.7 | Sulfaclozine | 284.72 | 0.93 |
| Sulfadiazine sodium | 272.26 | Sulfadiazine | 250.28 | 0.92 |
| Sulfamethoxazole sodium | 275.26 | Sulfamethoxazole | 253.28 | 0.92 |
| Sulfamonomethoxine sodium | 302.29 | Sulfamonomethoxine | 280.31 | 0.93 |
| Tilmicosin phosphate | 967.1 | Tilmicosin | 869.147 | 0.90 |
| Tylosin phosphate | 1014.1 | Tylosin | 916.1 | 0.90 |
| Tylosin tartrate | 1066.2 | Tylosin | 916.1 | 0.86 |

Kim, S., Chen, J., Cheng, T., Gindulyte, A., He, J., He, S., Li, Q., Shoemaker, B.A., Thiessen, P.A., Yu, B., Zaslavsky, L., Zhang, J., Bolton, E.E., 2023. PubChem 2023 update. Nucleic Acids Res. 51, D1373–D1380. https://doi.org/10.1093/nar/gkac956

**Table S2.** Summary of the used daily dose (UDD, mg/kg/d) per AMD active ingredient. All drugs listed in the table were administered orally (through feed and drinking water) and considered for the usage purpose (therapeutic and preventive). UDD values are given in mg/kg body weight. AMDs were listed based on different priority AMEG categorization defined by EMA, where “category B-restrict”, category C-caution”, and “category D-prudence”

| AMD (active ingredient) | Category | Therapeutic purpose | | Preventive purpose | |
| --- | --- | --- | --- | --- | --- |
|  |  | UDD median (IQR) | UDD (min-max) | UDD median (IQR) | UDD (min-max) |
| Colistin | B | 5.49 (1.08–16.70) | 0.40-89.49 | 2.21 (1.12–6.90) | 0.10-257.20 |
| Enrofloxacin | B | 51.82 (43.75–53.88) | 23.54-56.08 | 23.54 (16.61–33.71) | 2.62-106.93 |
| Norfloxacin | B | 47.43 (47.00–47.86) | 46.57-48.29 | - | - |
| Florfenicol | C | 21.38 (9.02–40.52) | 0.38-196.13 | 23.36 (15.01–38.38) | 4.18-277.02 |
| Tilmicosin | C | 20.55 (16.63–30.17) | 15.34-44.76 | 21.28 (20.35–30.24) | 11.08-46.80 |
| Tylosin | C | 11.81 (11.02–12.54) | 9.77-13.09 | 25.31 (6.99–109.32) | 6.48-149.59 |
| Neomycin | C | 9.14 (9.01–9.27) | 8.87-9.41 | 11.60 (1.44–17.03) | 1.29-24.75 |
| Gentamicin | C | 9.76 (9.59–9.95) | 9.41-10.14 | 13.42 (11.09–15.65) | 0.41-57.75 |
| Cefalexin | C | - | - | 28.37 (27.05–29.65) | 26.40-68.36 |
| Apramycin | C | - | - | 114.10 (108.97–119.73) | 103.84-125.36 |
| Azithromycin | C | - | - | 20.62 (18.65–23.21) | 17.05-26.63 |
| Ampicillin | D | 11.38 (8.93–20.42) | 3.53-33.37 | 10.47 (4.35–17.01) | 1.30-69.21 |
| Amoxicillin | D | 55.16 (38.20–73.22) | 3.59-417.07 | 23.63 (12.35–47.76) | 2.74-312.78 |
| Doxycycline | D | 22.41 (17.39–24.29) | 0.19-173.38 | 22.41 (9.16–36.75) | 1.67-243.24 |
| Sulfamonomethoxine (alone) | D | 44.13 (44.13–104.40) | 44.13-112.43 | 56.33 (43.42–65.19) | 18.47-104.02 |
| Sulfamonomethoxine (combination with trimethoprim) * | D | 35.97 (22.82–44.81) | 17.87-195.33 | 32.50 (28.34–34.77) | 6.18-82.51 |
| Sulfaclozine | D | 20.10 (7.21–20.45) | 6.94-20.85 | 29.15 (9.69–62.98) | 5.56-79.48 |
| Sulfadimidine | D | 18.95 (5.26–19.28) | 5.09-19.61 | 25.09 (19.90–28.16) | 10.37-31.60 |
| Trimethoprim | D | 9.49 (4.52–10.06) | 0.43-52.51 | 6.98 (4.76–9.22) | 1.29-20.63 |
| Sulfadiazine | D | - | - | 22.61 (6.44–23.46) | 6.43-24.33 |
| Sulfamethoxazole | D | 27.23 (22.79–34.62) | 16.86-35.79 | 33.41 (33.12–33.70) | 32.84-34.00 |
| Oxytetracycline | D | 10.59 (10.34–10.84) | 10.09-11.09 | 23.44 (22.92–59.96) | 6.80-100.04 |
| Sulfadimerazine | D | - | - | 52.42 (49.93–54.92) | 47.43-57.42 |
| Sulphaquinoxaline | D | 54.76 (53.12–56.62) | 51.49-58.48 | - | - |
| Sulphaguanidine | D | - | - | 4.50 (3.55–11.60) | 3.41-12.75 |
| Bacitracin | D | 25.91 (25.35–26.59) | 24.74-27.31 | 34.94 (34.51–35.37) | 34.11-35.86 |

*: Combination products of sulfonamides with trimethoprim.

**Table S3.** Summary of GAMM results of sensitivity analyses replacing same-day mortality with (i) previous-day mortality (lag 1) and (ii) mean mortality over the preceding 2 (lag 2) and 3 (lag 3) days

| Model | lag | Smoothing functions | edf | $\chi^{2}$ | p-value |
| --- | --- | --- | --- | --- | --- |
| Preventive AMU | Lag 1 | within-flock mortality (lag 1) | 1.80 | 0.39 | 0.86 |
|  | Lag 2 | within-flock mortality (lag 2) | 1.00 | 0.07 | 0.80 |
|  | Lag 3 | within-flock mortality (lag 3) | 1.00 | 0.30 | 0.58 |
|  | Lag 1 | age of broiler × within flock mortality (lag 1) | 1.40 | 1.46 | 0.52 |
|  | Lag 2 | age of broiler × within flock mortality (lag 2) | 1.44 | 2.46 | 0.36 |
|  | Lag 3 | age of broiler × within flock mortality (lag 3) | 1.78 | 4.54 | 0.12 |
| Therapeutic AMU | Lag 1 | within-flock mortality (lag 1) | 3.85 | 10.79 | 0.04 |
|  | Lag 2 | within-flock mortality (lag 2) | 3.68 | 13.78 | 0.01 |
|  | Lag 3 | within-flock mortality (lag 3) | 3.18 | 14.66 | 0.01 |
|  | Lag 1 | age of broiler × within flock mortality (lag 1) | 4.86 | 12.98 | 0.05 |
|  | Lag 2 | age of broiler × within flock mortality (lag 2) | 3.90 | 8.99 | 0.13 |
|  | Lag 3 | age of broiler × within flock mortality (lag 3) | 3.16 | 6.48 | 0.17 |
| AMU regardless of purpose | Lag 1 | within-flock mortality (lag 1) | 2.35 | 4.32 | 0.23 |
|  | Lag 2 | within-flock mortality (lag 2) | 4.33 | 14.43 | 0.02 |
|  | Lag 3 | within-flock mortality (lag 3) | 3.27 | 13.65 | 0.01 |
|  | Lag 1 | age of broiler × within flock mortality (lag 1) | 5.17 | 9.33 | 0.20 |
|  | Lag 2 | age of broiler × within flock mortality (lag 2) | 2.72 | 10.77 | 0.02 |
|  | Lag 3 | age of broiler × within flock mortality (lag 3) | 3.67 | 12.18 | 0.02 |

* EDF = effective degree of freedom, which measures the actual model complexity (wiggliness) of a smooth term. The EDF equals 1 if the smooth term is penalized to a simple linear relationship, while higher values indicate non-linear, more complex curves. Ref. DF = reference number of degrees of freedom used in computing test statistic and the p-values.

**Table S4**. Phase-specific slopes (posterior median [95% HDI]) for both preventive AMU model and therapeutic AMU model

| Preventive AMU | | | | | | | |
| --- | --- | --- | --- | --- | --- | --- | --- |
| Scenario | CP1 | CP2 | CP3 | phase1 | phase2 | phase3 | Phase4 |
| S0_P | 57 | 79 | - | 0.80 [0.75, 0.85] | 1.25 [1.04, 1.48] | 0.71 [0.61, 0.81] | - |
| S1_P | 56 | 78 | - | 0.79 [0.74, 0.84] | 1.27 [1.06, 1.51] | 0.70 [0.61, 0.80] | - |
| S2_P | 55 | 77 | - | 0.78 [0.73, 0.84] | 1.29 [1.08, 1.54] | 0.71 [0.61, 0.80] | - |
| S3_P | 54 | 76 | - | 0.77 [0.72, 0.83] | 1.32 [1.10, 1.56] | 0.71 [0.61, 0.80] | - |
| Therapeutic AMU | | | | | | | |
| S0_T | 27 | 53 | 83 | 1.28 [0.99, 1.63] | 0.68 [0.53, 0.84] | 1.35 [1.10, 1.64] | 0.57 [0.43, 0.71] |
| S1_T | 26 | 52 | 82 | 1.34 [1.01, 1.72] | 0.66 [0.52, 0.82] | 1.37 [1.11, 1.67] | 0.58 [0.44, 0.72] |
| S2_T | 25 | 51 | 81 | 1.39 [1.03, 1.81] | 0.65 [0.51, 0.81] | 1.38 [1.12, 1.68] | 0.60 [0.46, 0.73] |
| S3_T | 24 | 50 | 80 | 1.45 [1.04, 1.93] | 0.64 [0.49, 0.80] | 1.40 [1.14, 1.70] | 0.60 [0.47, 0.74] |

**Table S5.** Predicted probabilities of preventive and therapeutic AMU (posterior median [95% HDI]) for a typical farm at their respective peak days within each phase

| **Preventive AMU** | | | | | | | |
| --- | --- | --- | --- | --- | --- | --- | --- |
| Scenario | CP1 | CP2 | CP3 | Day 30 | Day 68 | Day 90 |  |
| S0_P | 57 | 79 | - | 0.04 [0.03, 0.05] | 0.02 [0.02, 0.03] | 0.02 [0.01, 0.03] | - |
| S1_P | 56 | 78 | - | 0.04 [0.03, 0.05] | 0.02 [0.02, 0.03] | 0.02 [0.01, 0.03] | - |
| S2_P | 55 | 77 | - | 0.04 [0.03, 0.05] | 0.02 [0.02, 0.03] | 0.02 [0.01, 0.02] | - |
| S3_P | 54 | 76 | - | 0.04 [0.03, 0.05] | 0.03 [0.02, 0.03] | 0.02 [0.01, 0.02] | - |
| **Therapeutic AMU** | | | | | | | |
|  |  |  |  | Day 13 | Day 40 | Day 68 | Day 122 |
| S0_T | 27 | 53 | 83 | 0.02 [0.01, 0.02] | 0.01 [0.01, 0.02] | 0.01 [0.01, 0.02] | 0.00 [0.00, 0.00] |
| S1_T | 26 | 52 | 82 | 0.02 [0.01, 0.02] | 0.01 [0.01, 0.02] | 0.01 [0.01, 0.02] | 0.00 [0.00, 0.00] |
| S2_T | 25 | 51 | 81 | 0.02 [0.01, 0.02] | 0.01 [0.01, 0.02] | 0.01 [0.01, 0.02] | 0.00 [0.00, 0.00] |
| S3_T | 24 | 50 | 80 | 0.02 [0.01, 0.03] | 0.01 [0.01, 0.02] | 0.01 [0.01, 0.02] | 0.00 [0.00, 0.00] |

**Table S6.** Summary of results of GEE-like model with AR(1) working correlation matrix, using the bam() function, with additive effects of within-flock mortality, broiler age, and their interaction, and the random intercept of the farm.

| Preventive  AMU | Parameters | Estimate | SE | p-value |
| --- | --- | --- | --- | --- |
|  | - intercept | -3.12 | 0.69 | <10^-6^ |
|  | Smoothing functions | Edf* | Ref.df* | p-value |
|  | - age of broiler | 4.43 | 5.63 | <10^-3^ |
|  | - within-flock mortality | 1.0 | 1.0 | 0.97 |
|  | - age of broiler $\times$ within flock mortality | 2.51 | 3.21 | 0.35 |
|  | - farm | 27.46 | 59 | <10^-6^ |
| Therapeutic  AMU | Parameters | Estimate | SE | p-value |
|  | - intercept | -3.6 | 0.54 | <10^-11^ |
|  | Smoothing functions | edf | Ref.df | p-value |
|  | - age of broiler | 3.03 | 3.67 | 0.23 |
|  | - within-flock mortality | 1.67 | 2.08 | 0.62 |
|  | - age of broiler $\times$ within flock mortality | 3.94 | 5.0 | 0.14 |
|  | - farm | 0 | 59 | 0.59 |
| AMU regardless of purpose | Parameters | Estimate | SE | p-value |
|  | - intercept | -2.73 | 0.53 | <10^-7^ |
|  | Smoothing functions | edf | Ref.df | p-value |
|  | - age of broiler | 5.0 | 5.49 | <10^-3^ |
|  | - within-flock mortality | 2.37 | 3.0 | 0.18 |
|  | - age of broiler $\times$ within flock mortality | 1.0 | 1.0 | 0.66 |
|  | - farm | 10.2 | 59 | 0.12 |

******* *EDF =* effective degree of freedom, which measures the actual model complexity (wiggliness) of a smooth term. A larger EDF implies more complex, wiggly splines (Wood, 2017). The EDF equals 1 if the smooth term is penalized to a simple linear relationship, while higher values indicate non-linear, more complex curves. Ref. DF = reference number of degrees of freedom used in computing test statistic and the p-values.
